# Identifying porous cage subsets in the Cambridge Structural Database using topological data analysis†

**DOI:** 10.1039/d2sc03171j

**Published:** 2022-10-31

**Authors:** Aurelia Li, Rocio Bueno-Perez, David Fairen-Jimenez

**Affiliations:** The Adsorption & Advanced Materials Laboratory (A^2^ML), Department of Chemical Engineering & Biotechnology, University of Cambridge Philippa Fawcett Drive Cambridge CB3 0AS UK df334@cam.ac.uk

## Abstract

As rationally designable materials, the variety and number of synthesised metal–organic cages (MOCs) and organic cages (OCs) are expected to grow in the Cambridge Structural Database (CSD). In this regard, two of the most important questions are, which structures are already present in the CSD and how can they be identified? Here, we present a cage mining methodology based on topological data analysis and a combination of supervised and unsupervised learning that led to the derivation of – to the best of our knowledge – the first and only MOC dataset of 1839 structures and the largest experimental OC dataset of 7736 cages, as of March 2022. We illustrate the use of such datasets with a high-throughput screening of MOCs and OCs for xenon/krypton separation, important gases in multiple industries, including healthcare.

## Abstract 2

We mined the Cambridge Structural Database for porous cages using topological data analysis, which resulted in the first and only dataset of metal-organic cages and the largest dataset of organic cages.

## Graphical abstract

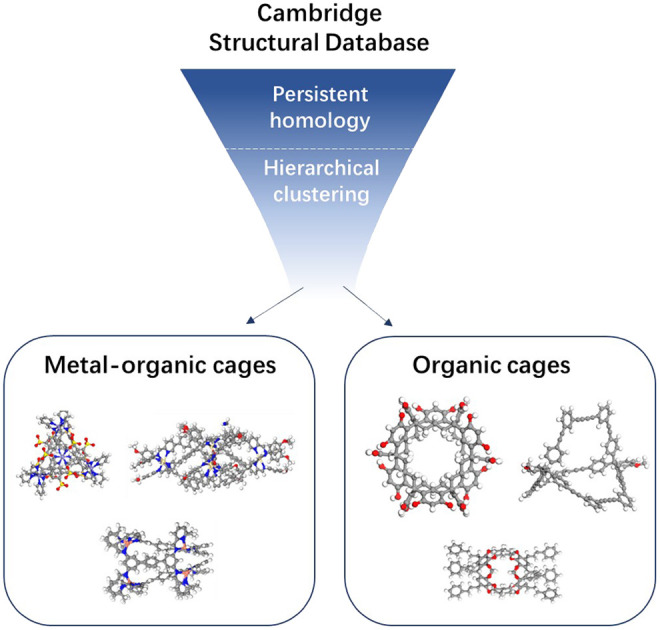

## 1. Introduction

Amongst the burgeoning field of microporous materials, metal–organic cages (MOCs) and organic cages (OCs) are of particular interest.^1–5^ Conversely to the more established metal–organic frameworks (MOFs) and covalent organic frameworks (COFs), which are extended crystalline structures constructed from strongly bonded building blocks, MOCs and OCs are discrete individual molecules with a cage-like shape. When packed, MOCs and OCs assemble through non-covalent interactions into a bigger porous structure. Therefore, there are two types of porosities to be considered with cages: (i) the molecule's internal cavity, called *intrinsic* porosity and (ii) the interstitial space due to packing, called *extrinsic* porosity. The combined porosities of these materials justify the growing research for their applications in molecular^6,7^ or gas separations,^1,2,6,8,9^ encapsulation,^10^ catalysis,^11,12^ molecular sensing,^5,13,14^ and as porous liquids.^15^ Although MOFs, COFs, MOCs and OCs all share some similarities – they are all tuneable porous materials, the discrete nature of MOCs and OCs means they are processable in solution. For instance, Giri *et al.* reported the preparation of ‘porous liquids’ by dissolving custom-designed rigid crown-ether cages in a solvent (15-crown-5) that cannot enter the cages.^15^ While methane is soluble in 15-crown-5 (6.7 μmol g^−1^ at 30 °C), adding the cages increased the solubility by eightfold (52 μmol g^−1^ at 30 °C). Cages can also be incorporated into polymers^16^ and form mixed-matrix membranes.^17^ Choosing between a MOC and an OC depends on the requirements of the application. For example, the open metal sites available in MOCs offer more tuning possibilities for catalysis, whereas OCs provide an overall lighter material.

Like in MOFs and COFs, the modularity of MOCs and OCs can lead to a large space of possible structures and has thus attracted the attention of computational researchers. The two types of porosities – intrinsic and extrinsic – add yet another customisable dimension. Evans *et al.* estimated that using only small organic molecules as building blocks for cage-based porous molecular materials, there could be 10^60^ potential candidates.^18^ Inspired by the success of high-throughput screenings (HTS) on extended porous materials, several research groups started to apply the same data mining methods to organic molecular materials, whether their porosity is intrinsic and/or extrinsic. McKeown *et al.* carried out a targeted structure search in the Cambridge Structural Database (CSD) to identify promising organic microporous crystals for nitrogen and hydrogen adsorption.^19^ In particular, the authors were looking for structures that might possess enhanced microporosity compared to existing examples of microporous crystals. Therefore, to narrow their search in the database, they looked for structures (1) with densities lower than 0.9 g cm^−3^, as the lowest density of any known microporous organic crystal was 0.96 g cm^−3^ for *p-tert*-butylcalix[4]dihydroquinone after water removal, (2) containing mostly aromatic rings as these play an essential role in the structures' stability (or shape persistence) and (3) with pore diameters smaller than 10 Å, as it was shown it ensures stronger gas adsorption for hydrogen storage. Following this data sieving and after the elimination of additional structures with questionable data quality, 23 organic and metal–organic candidates were retained. Among them, 3,3′,4,4′-tetra(trimethylsilylethynyl)biphenyl (CSD refcode: BALNIM^20^) was synthesised experimentally and demonstrated a BET (Brunauer–Emmett–Teller) surface area of 278 m^2^ g^−1^ and the highest amount of nitrogen adsorbed at 77 K and at saturation for an organic, crystalline compound with such low molecular mass.^20^ Later on, Mastalerz *et al.* used similar criteria to rationally build an extrinsically porous molecular crystal with flat-ordered sheets self-assembled with hydrogen bonding.^21^ They found that benzimidazolones were promising subunits for extrinsic porous crystalline structures with one-dimensional channels. The synthesised structure (a trisbenzimidazolone, CSD refcode: DEBXIT^21^) showed an exceptional BET area of 2796 m^2^ g^−1^.

Moving on from single searches to larger datasets, Evans *et al.* derived the first organic porous molecular crystals database (oPMC).^22^ From the CSD (version 5.35, including updates up to March 2014), the authors used ConQuest to look for (1) organic structures with (2) densities lower than 2 g cm^−3^, containing either (3) only one residue (therefore removing co-crystals) or (3′) more than one symmetry-independent molecule. In this search, they excluded (1) disordered structures, (2) structure data solved from powder diffraction methods, (3) a list of structure types including organic polymers, amino-acids, peptides and complexes. From the initially obtained dataset of 160 000 structures, entries without explicit hydrogen atoms were removed, leading to 156 333 candidates. Among these, 16 000 were found to be porous to helium. However, a significant number of structures presented unphysically large pores that could potentially lead to mechanically unstable structures and their collapse. To remove these, molecular mechanics simulations were performed to optimise the geometry of the crystals. 481 final organic porous molecular crystals were eventually retained to form the oPMC. These include well-studied structures as well as previously unknown ones. The authors also demonstrated the possible structure–properties trends identifiable with such a dataset. In particular, they applied support vector machines on oPMC to show that descriptors related to molecular sizes – such as van der Waals surface – are the most important factors in predicting the structures' propensity to form structural voids. While the previous studies did not specifically focus on intrinsically porous materials, Miklitz *et al.* built the Cage Database (CDB), which contains organic cages, cucurbiturils, cyclodextrins and cryptophanes, for xenon/krypton separation purposes specifically.^23^ Starting from about 120 structures identified through a literature review, 41 structures were first retained after visual inspection. 26 of them were then found to have pore sizes suitably close to the diameters of xenon and krypton, after which only the structures with the highest host–guest binding energies and the relative xenon/krypton binding energies were kept, leading to 12 potential candidates. Conversely to the previous studies, this screening focused on the analysis of single host molecules, rather than the solid-state structure of the material. The experimental adsorption isotherms of xenon and krypton were then measured for the selected materials at 1 bar and 298 K. They found that the cage molecule Covalent Cage 3 (CC3) remained the best-performing structure for this task, theoretically and experimentally, as previously reported.^6^ In an effort to map out the landscape of known porous organic cages, Sturluson *et al.* obtained a latent cage space by deriving “eigencages” from a dataset of 74 cages.^24^ These cages consist of the 41 structures from the CDB and 33 cages discovered and synthesised *via* high-throughput robotic synthesis.^25^ To obtain this latent representation, the cages were first scanned to obtain 3D images of their intrinsic porosity. Using singular value decomposition, the data corresponding to these 3D images were then compressed to obtain an approximate low-dimension subspace defined by an orthogonal basis composed of vectors called “eigencages”. Any cage (from this dataset or similar to the cages in this dataset) lies in this low-dimension subspace and can be described as a linear combination of these eigencages. The dimension of this latent space was chosen so as to minimise the loss of information during the compression step. From the 74 cages in this work, the authors derived a subspace of dimension 22, corresponding to a 70% compression and a 15% relative error when reconstructing the cages. Interestingly, of the 22 eigencages, the six most important ones are often enough to visually reconstruct the structure of a given cavity. Each eigencage, being each an eigenvector, gives a direction that explains the most variance in the 3D images. However, only the three most important eigencages captured human-understandable features of the cavity shape (such as cavity size and protrusion). The other eigencages were less intuitive and more difficult to interpret.

While the computational organic molecular materials field has significantly grown, the metal–organic equivalent seems currently non-existent. Yet, the field itself is growing, as demonstrated by the increasing number of reviews tackling a significantly diverse range of MOCs.^4,26–35^ Similarly to our previous efforts with MOFs and COFs subsets, armed with the CSD tools,^36,37^ we would like to answer the question: how many MOCs and OCs are there?

**Fig. 1:**
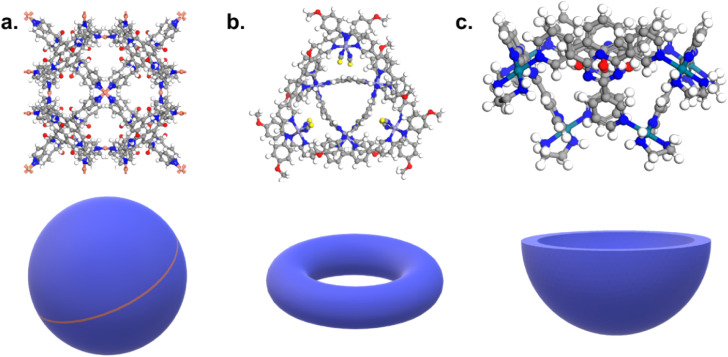
Examples of (a) a cage, (b) a ring-like structure, (c) a bowl-shaped structure. CSD refcodes: (a) CIYWOX,^39^ (b) AVELIYH,^40^ (c) BOGYUT.^41^ 3D schematic of the corresponding shapes are provided at the bottom of each example in blue.

Conversely to MOFs and COFs, however, cages are discrete molecules. Extended structures were identified in the CSD using a combination of (i) a substructure search based on the most common ligands and clusters linkages and (ii) a search for keywords tagging “polymeric” structures specifically. The latter captured the essence of extended structures and significantly reduced the search space for (i). However, simply changing the second criterion to “non-polymeric” structures significantly enlarges our search area without getting us any closer to cage-shaped molecules. Indeed, a search for non-polymeric organo-metallic structures with 3D coordinates determined leads to 447 336 hits, and the same search for organic structures returns 442 503 candidates (CSD version 5.41 with updates up to November 2019). While the specific linkage between the organic and metal subunits can still be described with ConQuest, there is no simple specific keyword to capture the shape of cages. In addition, the lack of a clear definition of cages makes their automatic identification even more difficult. The IUPAC defines cage compounds as “polycyclic compound[s] having the shape of a cage”,^38^ which, similarly to the MOFs' case,^36^ translated into yet another tautology. While it seems widely assumed and accepted that cages should contain cavities, it remains unclear when a cage should no longer be considered a cage: how closed or open can these cavities be, in order to be labelled as a cage? While certain structures are undoubtedly cages (3D) and other rings (2D), there is a wide range of structures in between these two extremes. Fig. 1 shows an example of each. What is certain is that cages, from a structural point of view – whether 3D or 2D – should contain some kind of hole in which another molecule can fit, at least partially.

**Fig. 2:**
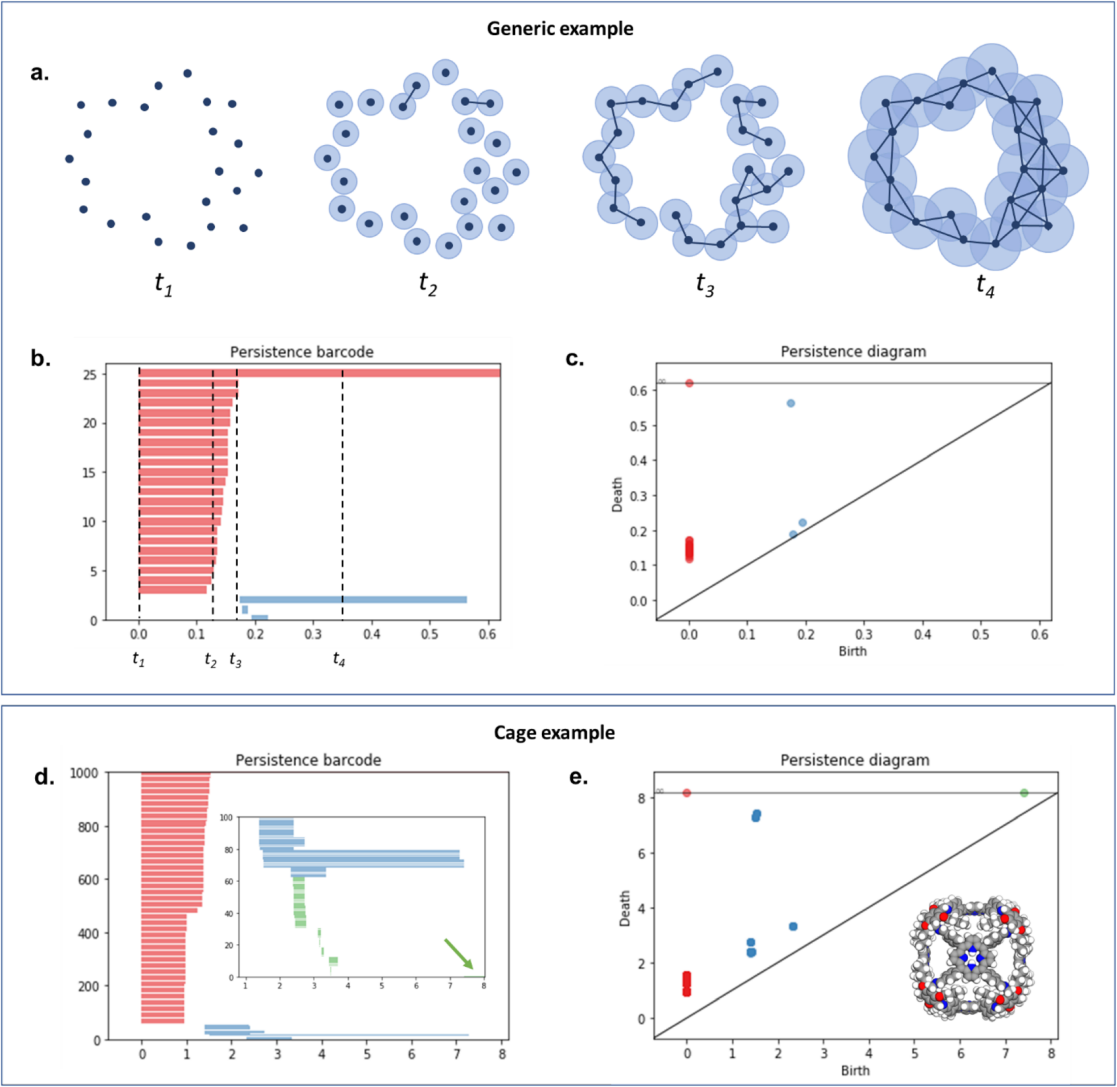
An illustration of persistent homology. Upper box: a generic 2D hole example. (a) The process of building a filtration on top of the set of points. The data points are represented with dark blue dots (*t*_1_). Light blue balls are then placed on top of each data point. The balls' radius is then gradually increased. When two balls meet, the corresponding data points are connected *via* a segment, forming a new connected component (*t*_2_). This process can also lead to the formation of triangles (*t*_3_). At *t*_4_, there is only one connected component left. The corresponding persistence barcode and persistence diagram are given in (b) and (c), respectively (red lines and dots: *B*_0_ components, blue lines and dots: *B*_1_ components). The time steps *t*_1_ to *t*_4_ are indicated in (b) for reference. Lower box: example of a cage (CSD refcode QUFYIB^44^). An image of the structure is provided in (e) the persistence barcode and diagram are given in (d and e), respectively (red lines and dots: *B*_0_ components, blue lines and dots: *B*_1_ components, green lines and dots: *B*_2_ components). A zoom on the *B*_2_ components is giving in the inset in (d).

To capture the presence of this hole – cavity in 3D or window in 2D, we chose to use a well-established data analysis tool called topological data analysis (TDA).^42^ TDA is a mathematical method that studies the shape – or topology, in mathematical terms – of big data. In particular, persistent homology^43^ enables the identification of holes and clusters of data points. We provide here a high-level description of persistent homology and leave the curious reader with the mathematical details. Persistent homology identifies the features that are the most spatially representative in a point cloud. For this, a nested family of *simplicial complex* is gradually built on top of the studied set of points. This simplicial complex consists of a set of points (0-simplex), line segments (1-simplex), triangles (2-simplex, in 2D) and tetrahedrons (3-simplex, in 3D) that form a graph where the segments, triangles and tetrahedra represent the relationships between the points. This family of simplicial complex is called *filtration*. Fig. 2a illustrates how this filtration is algorithmically obtained for a 2D point cloud. Given an appropriate metric distance (in our case, the Euclidean distance to study the spatial relationship of atoms in a molecule), a ball (or sphere in 3D) of a given radius is virtually centered on top of each of the data points at the starting time *t* = 0. Fig. 2a shows the data points at *t*_1_ = *t*_0_^−^, just before the balls are placed on top. We then increase the balls' radius. When two balls meet each other, a segment between the two corresponding data points is created (see *t*_2_ in Fig. 2a). *t*_3_ in Fig. 2a shows a time step when a triangle is created from three neighbouring data points. As the balls grow in radius and merge, a more complete filtration is gradually obtained. Visually, at *t*_4_, the balls and filtration have already captured the presence of a hole formed by the data points. The algorithm can continue until the balls have all merged and covered the hole; this is when the full filtration is obtained. A useful concept in persistent homology is the Betti number. The *k*th Betti number *B*_*k*_ is the number of independent *k*-cycles that are not the boundary of an object of dimension *k* + 1. Here, *k*-cycles are linear combinations of *k*-simplices with an empty boundary, *i.e.* these linear combinations potentially surround a region of *k* + 1 dimension. The easiest to visualise are 1-cycles: they are loops, such as the three edges (and boundary) of a triangle. Simply put, *B*_0_ is the number of independent connected components, *B*_1_ the number of holes and *B*_2_ the number of cavities. During the computation of the filtration, two important time steps are recorded for each component: their birth time and their death time. The birth time of a component corresponds to the time it is first created. At *t* = 0, the balls of radius 0 placed on top of each data point represent each data point as a single component. When two data points are connected *via* a segment, the initial two components have “died” and a new component (the two points and the connecting segment) is created. This marks the death of the first two components and the birth of the new component.

From the recorded births and deaths, it is then possible to represent the persistence homology with the help of persistence diagrams and persistence barcodes. In the latter, each individual component at any given time is stacked on the *y*-axis, in order of successive births. Their lifetime is represented on the *x*-axis by joining the components' birth time and death time. The components corresponding to different Betti numbers are represented in different colours. The number of most persistent components at the final time gives the number of independent components. In the persistence diagram, the births are recorded on the *x*-axis and the deaths on the *y*-axis. Each component is then represented by a single point, above the diagonal line. Fig. 2b and c show the persistence barcode and diagram obtained for the 2D point cloud presented in Fig. 2a. The red lines in the barcode and the red dots in the diagram correspond to *B*_0_ components. At the start (*t*_1_), each data point corresponds to a single independent component. Hence, a large number of red lines are stacked on top of each other. These independent components quickly give way to a smaller number of connected components of a higher Betti number. The blue lines and dots correspond to *B*_1_ components. The longest blue line corresponds to the most persistent component, *i.e.* the hole. This long blue line coexists with a long red line, meaning it is also the only component left. The corresponding point in the persistence diagram is the blue dot high above the diagonal, at time *t* = 0.2. Fig. 2d and e give an example of the persistence diagram and barcodes obtained for a cage (see image of the structure in Fig. 2e). The stack of red lines (*B*_0_) is very tall because of the high number of atoms. A few blue lines (*B*_1_) are significantly longer than other blue lines – they correspond to the blue points located at death times equal to about 1.5, high above the diagonal. They signal the presence of large windows. The green lines and green points (see the zoom in the inset of Fig. 2d) correspond to *B*_2_ components. Importantly, the most persistent component is indicated by the only long green line left at the end of the calculation (indicated by a green arrow). This green line is translated into one distinct green point at a death time of about 7, and well above the diagonal. This point corresponds to the cavity.

**Fig. 3:**
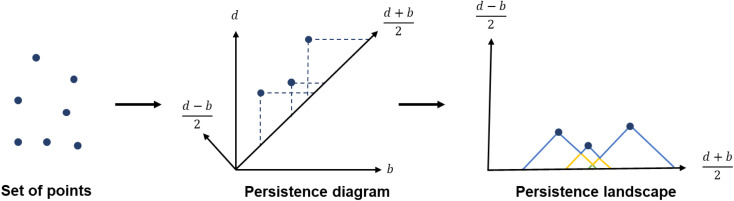
From data points to persistence diagram to persistence landscape.

While persistence diagrams and barcodes are the most intuitive representations of persistence homology, they are often not readily useable for further comparative data analysis, as each structure has a different number of (birth, death) points. This is when persistence landscapes come in handy.^45^ A persistence landscape takes as input the previously obtained persistence diagram and turns it into a set of functions. Fig. 3 illustrates it. It is then possible to choose a fixed number of points from this set of functions to represent a given set of structures. Putting the chosen points into vectors of same lengths allows us to then apply machine learning to all the structures.

Persistent homology has been applied in the field of nanoporous materials on several occasions. Lee *et al.* used TDA to analyse the pore shapes of zeolites and their impact on the adsorption performances.^46^ Later on, TDA-based descriptors were used to predict with machine learning the performance of structures of similar shapes.^47^ Moosavi *et al.* used persistent homology to define the geometry landscapes of porous molecular crystals from the crystal structure prediction datasets.^48^ In their work, they choose three molecules and studied the shapes of their various packings. The different types of packings identified were mapped to their corresponding lattice energies, thereby revealing the best-performing structures. Machine learning was then applied, using these geometric landscapes as descriptors, and performed remarkably well for the prediction of methane storage.

## 2. Cage identification

Here, we aimed to apply machine learning to the persistent homology fingerprints obtained to predict whether the candidate structures are cages or not. Similarly to the CSD MOFs subset, we chose to identify both 2D (rings) and 3D (cages) structures in order to keep the dataset useful to the widest audience possible. It is important to note that we are focused on identifying the presence of a single molecule with a cavity or windows, and not on the periodic structure obtained from their packing. Although important and essential to understanding their adsorption behaviour, the extrinsic porosity of MOCs and OCs is beyond the scope of this study.

**Fig. 4:**
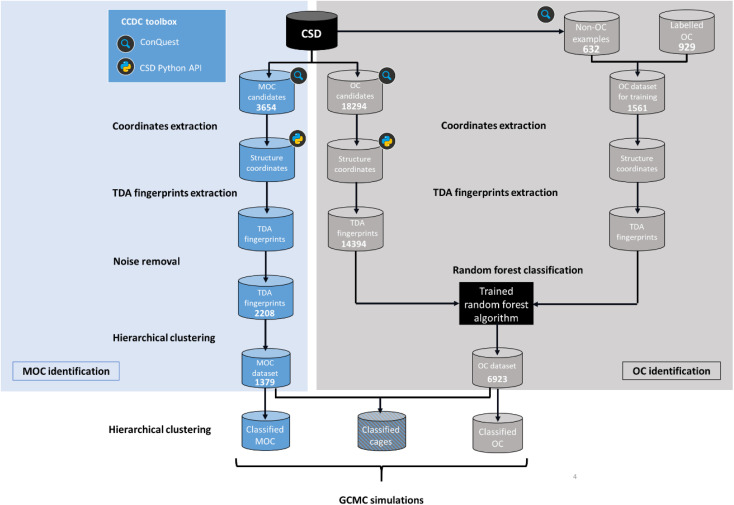
Workflow for the identification and classification of metal–organic cages (MOCs) and organic cages (OCs) in the Cambridge Structural Database (CSD).

While the problem for MOCs and OCs is the same – identifying their defining shapes – our starting point for these two types of structures is different. As previously explained, there are no known datasets of experimental MOCs in the literature, whereas some experimental OCs have been extracted already.^23^ In particular, we used in this work a list of known experimental OCs kindly provided by the Jelfs Computational Materials Group and available at https://github.com/andrewtarzia/cage_datasets. This existing dataset of OCs was obtained by looking for known author names in the CSD and consists of 929 2D and 3D structures. Fig. 4 shows the two distinct workflows we used for MOCs and OCs. While MOCs underwent unsupervised classification, OCs were determined with supervised classification. In addition, the large amount of discrete organic or metal–organic structures in the CSD encouraged us to reduce our search space and computational time by first carrying out ConQuest searches for potential MOCs and OCs candidates. We first used the CSD version 5.41 with updates up to November 2019 to demonstrate our method. Once a list of potential candidates was obtained, the structures were further processed with the CSD Python API:

• Many entries have either additional solvent molecules or multiple identical cages. Both cases add unnecessary noise to the TDA analysis, and only the heaviest weight component (heaviest_component in the CSD Python API) corresponding to the cage of interest was kept. This also helps us identify the *single* cage we want to extract the coordinates from.

• Although rare, some entries are fully linear. Therefore, an additional check made sure that at least one atom is part of a cycle. Note that ‘cycle’ here includes any closed path from a given atom to the same atom.

We then extracted the fractional coordinates of the cleaned isolated molecules, allowing us to perform TDA on a single molecule, and not on the full periodic structure. We used the Python GUDHI module for the TDA calculations.^49^ We used the Vietoris–Rips complex to build the simplicial complex. This complex is a set of points built such that the distance between two points is less or equal to a given alpha (Fig. S2†). The maximum value of alpha (max_edge_length) is provided by the user. To choose max_edge_length, several values ranging from 0.2 to 1.6 were attempted on randomly selected structures. When comparing the resulting persistences, we found that a large maximum value such as 1.6 considerably slows down the computation of the complex, while a low value such as 0.2 shows a more significant difference from the persistences obtained with higher values. Any value between these two extremes returned identical results and 0.8 proved to be a good middle-ground. We then computed the persistence landscapes. Since we are interested in the Betti 1 (windows) and Betti 2 (cavities) features, we obtained landscapes for each of these two dimensions for each structure. 2500 points were then sampled from each of the Betti 1 and Betti 2 landscapes, totalling 5000 fingerprint points. More details specific to each type of structures are given below. In the case of MOCs, an additional noise removal step was added to the workflow. As we explain later, we found some noisy structures, whose presence hampered the clustering algorithm. All the Python scripts used here are available on Github (https://github.com/ayl23/TDA_cages).

### 2.1 Metal–organic cages

#### 2.1.1 Data preparation

**Fig. 5:**
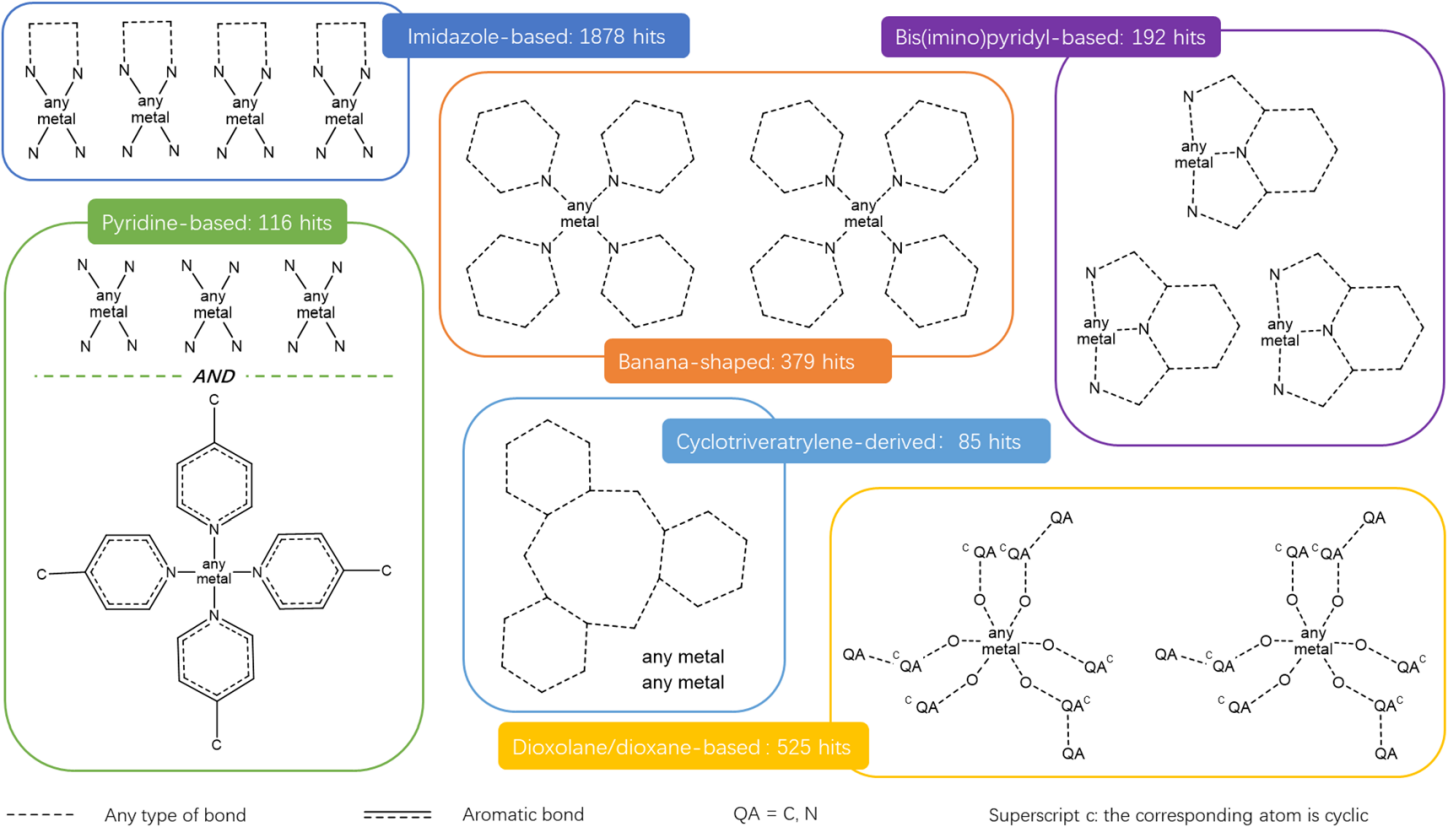
Quick ‘must-have’ criteria drawn in ConQuest for some common cages. The dotted lines refer to ‘Any’ type of bonds. QA = C or N. Upperscript c: the corresponding atom should be cyclic. When atoms are not explicitly indicated, they correspond to C atoms.

We identified some of the most common types of cages synthesised by, arguably, the largest MOC groups (Ward, Hardie, Clever, Nitschke, Raymond, and Fujita)^35,50–55^ and built simple ‘must-have’ criteria describing the linkage between their organic and metal parts with ConQuest. Six main groups were identified, of which the corresponding descriptions are provided in Section S2 of the ESI.† As the goal of these criteria is only to reduce the search space, they were not fine-tuned to match specific cages. Fig. 5 gives a summary of the different linkages, criteria and hits obtained. These criteria represent fragments of the targeted structure and are drawn in the *Draw* section of ConQuest to form a search query.^56,57^ This query is then translated to a CCDC-specific query format that encodes both the chemistry, the connectivity of the input substructure and any geometrical constraints added by the user (*e.g.* distance between atoms, number of connected atoms). The software then performs a graph-based search by comparing the queried substructure with all the other CSD entries. In addition to these criteria, the filters *3D coordinates determined*, *not polymeric* and *only organometallics* were used. As it was pointed out to us during the peer review process, carboxylate-based cages have been left out of Fig. 5. We take this opportunity to demonstrate at the end of our paper how other types of structures can be easily included using our method.

**Fig. 6:**
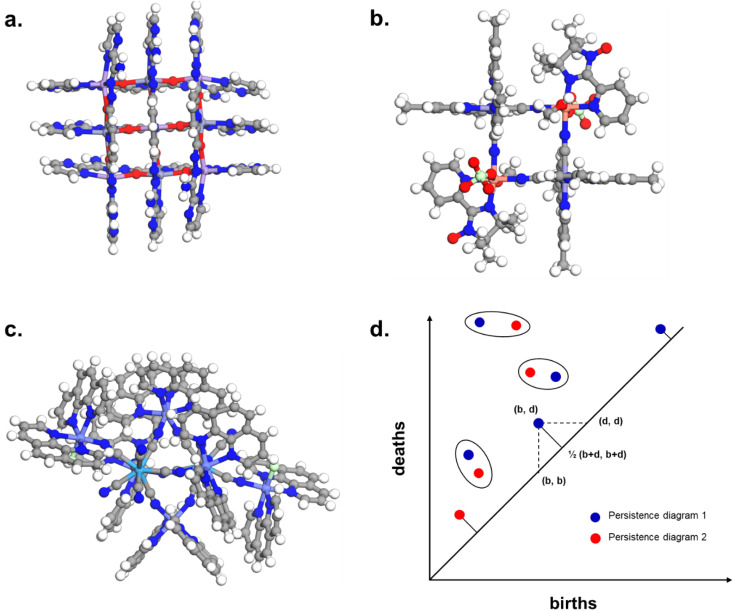
Removing noisy structures. CSD refcodes of example noisy structures: (a) BOYJOP,^58^ (b) AHABOA^59^ and (c) AGAPAA,^60^ (d) illustration of the calculation of the bottleneck distance between two persistence diagrams. The blue dots and the red dots come from two different persistence diagrams. The circles represent dots from the two persistence diagrams that have been matched, and on which the sup norm will be applied. For points that have not been matched, it is the sup norm to the closest point on the diagonal that is taken into account.

The combination of the presented queries led to a total of 3654 structures. Visual checks revealed a large amount of questionable structures such as AHABOA and BOYJOP (Fig. 6a and b). These structures have the shape of single and quadruple grids, respectively, in addition to being in large numbers. Structures in the shape of AGAPAA (Fig. 6c) are also in large numbers. Although these structures are usually not labelled as cages in the literature, they qualify structurally – mathematically – as cages. We obtained their persistence landscapes and, after a preliminary unsupervised classification of the candidates including these unusual structures, we found the latter confused the algorithm and reduced the overall classification performance. We, therefore, proceeded to remove these structures and, here on, refer to them as noise.

#### 2.1.2 Noise removal

Given two persistence diagrams, it is possible to compute their similarity. Several different measures of similarity exist. In this work, we used the standard bottleneck distance *d*_b_. To compute *d*_b_ we first pair up points from the two diagrams. Because the number of points is not necessarily the same in the two diagrams, points that do not have matched points from the other diagram are paired up with their closest point on the diagonal. Fig. 6d illustrates the calculation of the bottleneck distance. The blue dots and the red dots represent two different persistence diagrams. The circles highlight points that have been matched. For a given point of coordinates (*b*, *d*), their closest point on the diagonal has coordinates 1/2(*b* + *d*, *b* + *d*). We then compute the distance between the matched points, and between the unmatched points and their closest point on the diagonal. The distance metric used for the bottleneck distance is the sup norm in 
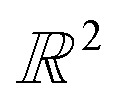
. The largest sup norm obtained constitutes the cost of the matching. However, there can be different possible matchings, each with a different cost. The final bottleneck distance is the smallest of all the possible costs, *i.e.* the cost of the most efficient matching. Section S1 of the ESI† provides some more mathematical details of the bottleneck distance calculation. We removed structures similar by 95% to the three identified noisy structures using the GUDHI module. We also observed that a number of structures did not contain an organic part in their main component. These structures were discarded using ConQuest, which left us with 2194 structures.

#### 2.1.3 MOC identification

**Fig. 7:**
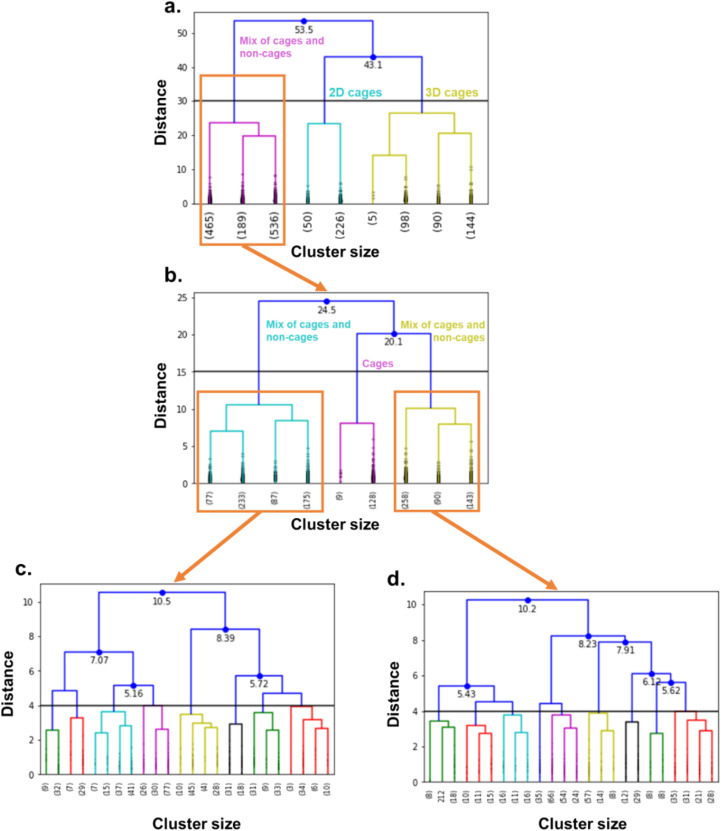
Truncated dendrograms of the hierarchical clusterings used to identify metal–organic cages. (a) Is the full dendrogram, (b) is the dendrogram obtained by zooming on the orange-highlighted area in (a), (c) is the zoomed-in dendrogram of the left orange-highlighted area in (b), and (d) is the zoomed-in dendrogram of the right orange-highlighted area in (b). The black horizontal line indicates interesting cut-off distance values.

We then applied hierarchical clustering on the persistence landscapes of the filtered structures. Hierarchical clustering is a type of unsupervised classification algorithm.^61^ In our work, we used agglomerative hierarchical clustering, where the initial clusters correspond to each different data point and are then merged together successively according to a specific merge strategy. The process resembles the building of a nested tree, where each branch corresponds to two merged clusters. That is why the final hierarchy of the clusters are represented as a tree – or dendrogram, where the root of the tree corresponds to the overall cluster containing all the structures. The obtained dendrogram is a useful way of visualising the similarity and relationships between the clusters and is the reason why this algorithm was chosen for this classification. We used the hierarchy, cluster and distance modules from the open-source Python library SciPy^62^ to compute the dendrograms on the 5000 fingerprint points sampled from the persistence landscapes. The chosen merge strategy was the standard Ward linkage, which defines the distance between two clusters as the variance between them and attempts to minimise it. Fig. 7a presents the first dendrogram obtained. The *x*-axis shows the different clusters obtained, and the number of structures in each cluster. For readability reasons, the dendrograms are truncated, therefore, showing only a small number of possible clusters. The *y*-axis represents the distance between each cluster merge, as calculated by the Ward method. The horizontal black line indicates interesting cut-off distances and guides users in choosing the number of desired clusters. Above this line, the distance at which two clusters merge is indicated in blue near the merging point. This first dendrogram is composed of three main branches. Visual checks of the classification show that the algorithm was able to clearly classify 2D and 3D cages (two right branches), with very few cases of non-cages. The first branch, however, is a mix of cages and non-cages. To obtain a closer and clearer view of this branch, we zoomed in by applying hierarchical clustering again. The resulting dendrogram is presented in Fig. 7b. There are again three main branches, the middle one being composed of 2D and 3D cages. The outer branches are however still composed of a mix of cages and non-cages. To analyse the structures in these branches, we zoomed in again and classified these branches into 48 clusters. The corresponding dendrograms are presented in Fig. 7c and d and truncated at 24 clusters for readability. Each of the smaller clusters was then visually checked to determine the ground truth for each structure. For each cluster, if the number of cages was higher than 60%, the whole cluster was considered as composed of cages. However, if less than 40% consists of cages, the whole cluster was considered as composed of non-cages. Using this decision method, we were able to estimate the accuracy of the overall method to be 94.7% for the 1377 structures labelled as cages. 112 structures were not classified, as they belonged to clusters with similar numbers of cages and non-cages. Among the structures labelled as non-cages, 26 were false negatives.

### 2.2 Organic cages

#### 2.2.1 Data preparation

**Fig. 8:**
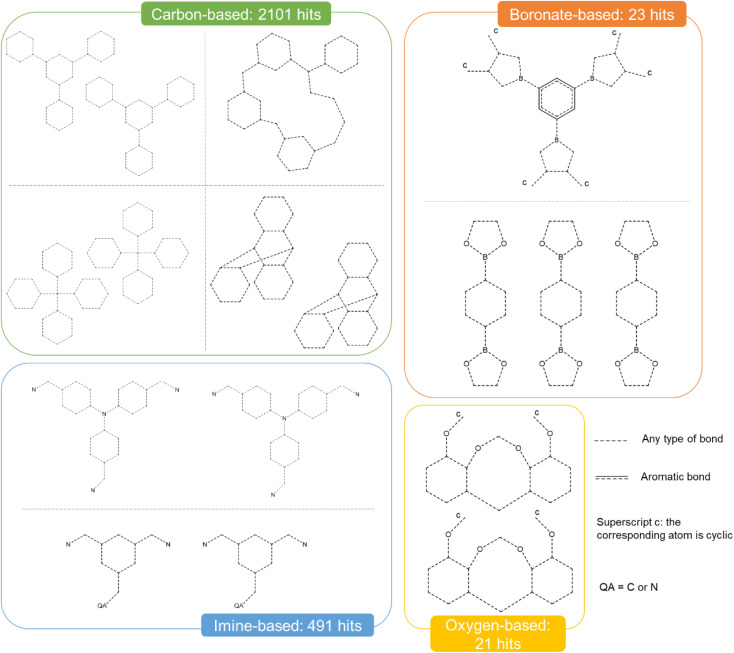
Quick ‘must-have’ criteria drawn in ConQuest for some common 3D organic cages. The dotted lines refer to ‘Any’ type of bonds. QA = C or N. Upperscript c: the corresponding atom should be cyclic. When atoms are not explicitly indicated, they correspond to C atoms.

Similarly to MOCs, we gathered potential OC candidates using ConQuest. A few main families of 2D and 3D cages were first identified based on literature reviews.^2,5^ Quick general queries were then designed to capture most of them, without any fine-tuning. The filters *3D coordinates determined*, *not polymeric* and *only organics* were used. In addition, we eliminated any structure with any metal atom. Fig. 8 summarises the main groups of organic cages and their respective number of hits. Examples of each type of OCs are given in Section S3 of the ESI.†

**Fig. 9:**
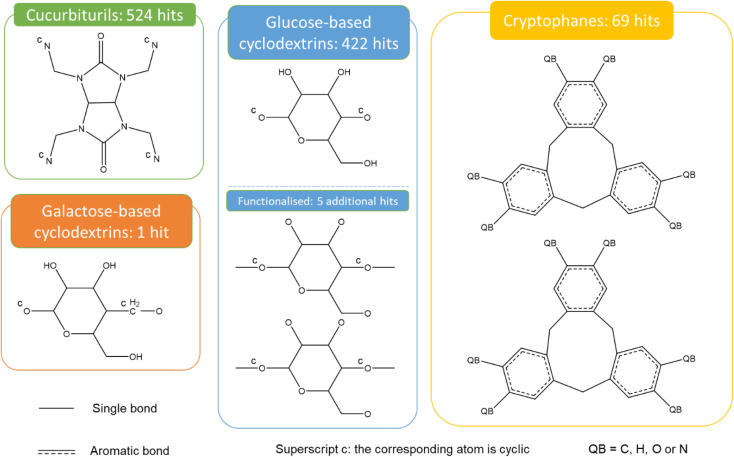
Quick ‘must-have’ criteria drawn in ConQuest for cucurbiturils, cyclodextrins and cryptophanes. The dotted lines refer to ‘Any’ type of bonds. QA = C or N. Upperscript c: the corresponding atom should be cyclic. When atoms are not explicitly indicated, they correspond to C atoms.

Fig. 9 presents the most common groups of rings considered and the corresponding number of hits returned: cucurbiturils, cyclodextrins and cryptophanes. The combination of the above queries led to a total of 3746 structures. When compared with the list of 929 labelled cages provided by the Jelfs Computational Materials Group, it was found that 462 structures were not found in the previous search. Although in minority, visual inspection showed these missing structures represented a wide variety of cages, not corresponding to any of the previously identified big families of OCs. For each type of missing structure, an additional query was created, until all missing structures were found. These queries are provided in Section S4 of the ESI.† A total number of 12 310 candidates was obtained, all of which had their persistent homology diagrams calculated.

#### 2.2.2 Supervised classification

To prepare the training dataset, we added to the list of structures labelled as cages 633 random non-cage structures from the CSD. These structures were visually checked to be indeed non-cages. We then obtained the persistent landscapes, as previously, and extracted a total of 5000 fingerprint points. The latter were fed to the RandomForestClassifier module from the Python library Scikit-learn^63^ to perform the supervised classification of the OCs. The base random forest model with 100 estimators was found to be good enough for the task. 85% of the data was used for training and 15% for testing. The trained algorithm had an accuracy of 96%. Among the 12 310 potential candidates, 6923 structures were labelled as cages, which is 7 times the size of the initial 929 labelled OCs.

## 3. Xenon/krypton separation

We have built here so far two CSD subsets of cages and showcased in particular the use of hierarchical clustering as an unsupervised classification method. This algorithm can be applied again on these two subsets – independently or jointly – to classify the different types of cages. To demonstrate the usefulness of such methods when mapped together with adsorption data, we carried out a HTS of a 20/80 xenon/krypton mixture at 298 K and 10 bar on the two datasets.

Separating xenon from krypton is of great industrial interest. Both are naturally found in low concentrations in the atmosphere: xenon is at 0.087 parts per million by volume (ppmv), and krypton at 1.14 ppmv.^64^ Both are also important in a range of applications, such as medical imaging,^65^ anaesthetics,^65,66^ lighting,^67^ lasers,^68^ double-glazing,^68^ and satellite propellant.^69^ At the moment, xenon/krypton mixtures are first obtained in a 20/80 ratio during cryogenic distillations performed during the separation of oxygen and nitrogen in the air.^70,71^ It is then necessary to apply additional cryogenic technologies to further purify the obtained xenon and krypton. Given the initial low concentrations, high-purity xenon is, therefore, as costly as 5000 USD per kilogram.^67^ A potentially cheaper alternative would be the use of selective adsorption in porous materials.

Several computational studies have already looked at the use of porous materials for xenon/krypton separation at 298 K.^6,72–76^ In particular, Simon *et al.* screened the nanoporous materials genome, composed of over 670 000 hypothetical and experimental zeolites, MOFs, COFs and other extended structures and found SBMOF-1 ^77^ to be a top MOF performer at 1 bar.^70^ Banerjee *et al.*, later on, screened 125 000 hypothetical and experimental MOFs and identified the very same SBMOF-1 in the same conditions.^74^ On the discrete molecules side, the tetrahedral organic cage CC3 was identified twice as the best performer at 298 K and 1 bar. These studies highlight, in particular, the importance of pore size and morphology with the selectivity of the material.^6,70,76^ Importantly, xenon's van der Waals radius is 1.985 Å, which is larger than that of krypton (1.83 Å).^70^ Combined with a deeper potential well for xenon, most structures are expected to be selective towards xenon. Sturluson *et al.* analysed their latent cage space by computing the excess uptake of xenon and krypton in a single cage molecule.^24^ They found that the shape of the cavity alone is a good indicator of the cavity's xenon/krypton selectivity, and cages that clustered in the same areas in the latent space have similar molecule and cavity sizes and show similar xenon/krypton selectivities. However, this study does not take into account the effect of packing on selectivities. While the current record-holder is well-established (SBMOF-1 has a predicted selectivity of 82 *versus* 13.8 for CC3),^70^ we show here how the cages classification can be mapped onto their separation performance. We specifically look at the cages' performance at 298 K and 1 and 10 bar.

### 3.1 Data preparation

We performed high-throughput grand canonical Monte Carlo simulations (GCMC, see methods in Section S5 of the ESI†) on the 1377 MOCs and 6923 OCs identified here and considered rigid. Note that, while the TDA was performed on single cages, the simulations are performed on packed cages. We removed the solvents using the same method as previously described for TDA, by using the CSD Python API to only keep the heaviest_component in each entry. For the cages, we used an atomistic model where the atoms were kept fixed at their positions and a mix of Dreiding force field^78^ and universal force field^79^ parameters. The xenon and krypton atoms were modelled with TraPPE. We found that many structures are tagged as “disordered” in the CSD. However, most of the disorders encountered in these structures are located in the solvent molecules. Therefore, by using the previous processing used to prepare the cages for TDA, we actually removed the disorder in most of the structures. Visual checking revealed a few structures presented missing hydrogens. We ran a bash script previously published to identify these structures,^37^ and used the CSD Python API's add_hydrogen() function to add missing hydrogens. The list of the structures identified is included on our Github page and we encourage the users to flag any other structures with missing hydrogens that the algorithm might have missed. The processing used in TDA kept only one single cage in the rare cases where multiple cages were present in one asymmetric unit. We, therefore, checked *a posteriori* that the well-ranked structures indeed only contained a single cage in one asymmetric unit.

We then applied hierarchical clustering on the two datasets, to further classify the cages according to their shapes and to determine any potential shape-selectivity relationships. We computed the dendrograms, visually identified interesting cut-off distances and chose the corresponding number of clusters accordingly. We chose 16 clusters for the MOC dataset and 11 clusters for the OC dataset.

### 3.2 Mapping the cages' shapes to their xenon/krypton separation performance

**Fig. 10:**
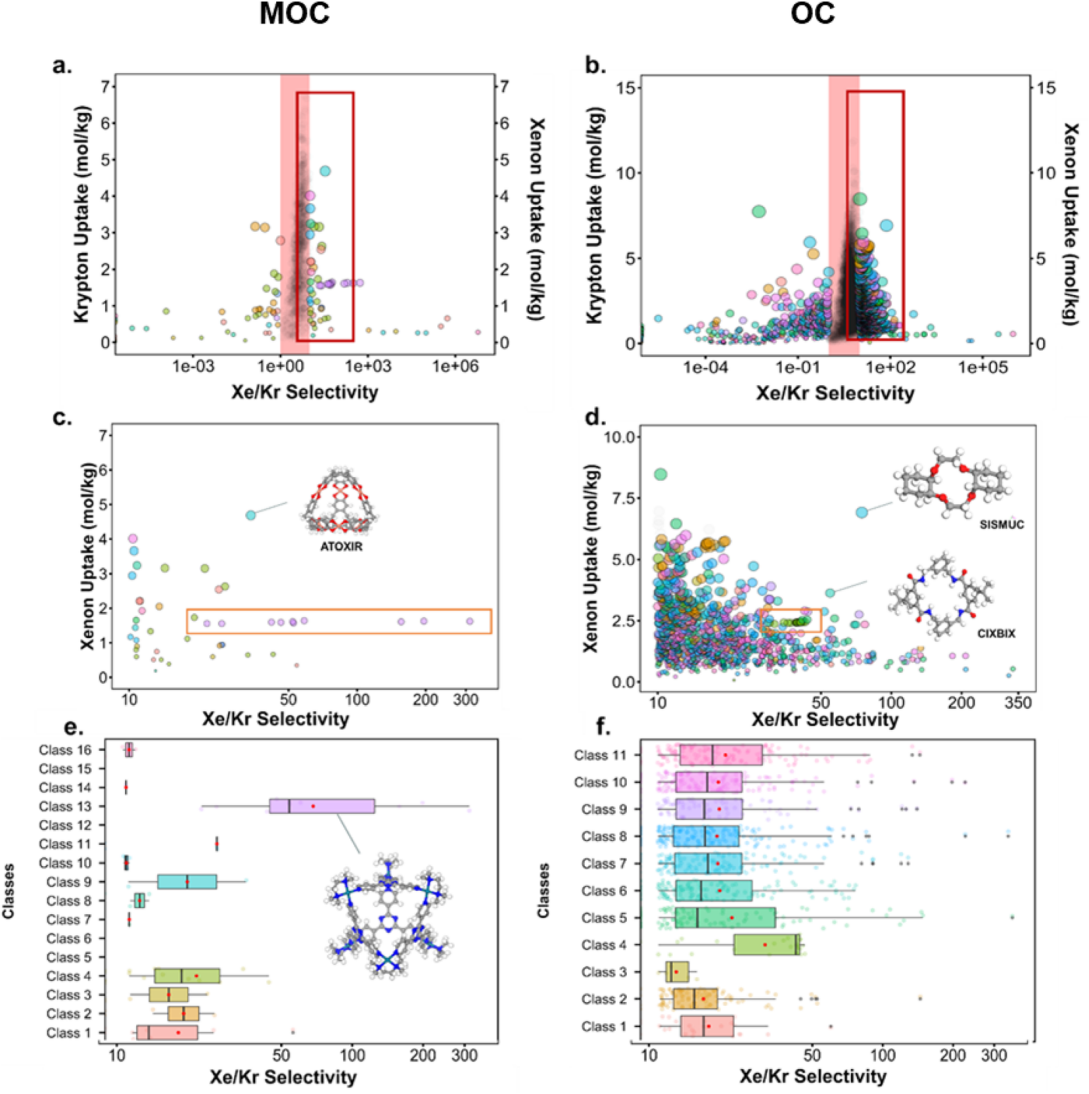
Xenon/krypton separation performance of metal–organic cages and organic cages. (a and b) Xenon and krypton uptakes *versus* Xe/Kr selectivity. Each point represents a structure. The points' colour corresponds to their cluster, indicated with the same colours in (e and f) the pink band hides structures with selectivities between 1 and 10. On the right side of the pink band, the *y*-axis and the size of the points correspond to the xenon uptake. On the left side of the band, the *y*-axis and size of the points correspond to the krypton uptake. The red boxes highlight areas of interest, zoomed in (c and d) the orange boxes indicate structures with a CC3-type shape. Images of the best-performing structures are also provided. (e and f) Show the boxplots of the different classes of materials identified for xenon/krypton selectivities of over 10. The jittered points in the background give an idea of the number of structures considered for each boxplot. The markers represent the minimum, first quartile, median, third quartile, and maximum values, respectively. The red dot indicates the mean. Outliers are represented by black data points.

Fig. 10 presents the results obtained by combining the GCMC results with the cages classification. The figures in the left column correspond to the MOC dataset and the figures in the right to the OC dataset. Fig. 10a and b present the xenon uptake *versus* the selectivity of xenon over krypton, here defined as:
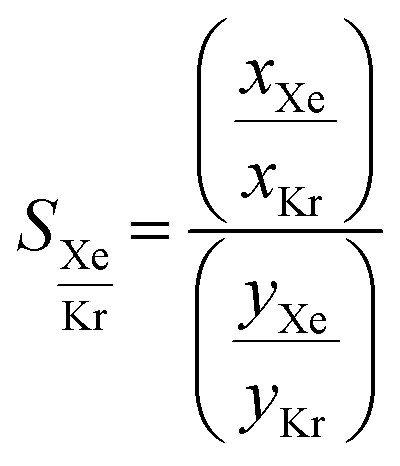
where *x*_Xe_ and *x*_Kr_ are the molar fractions of xenon and krypton in the adsorbed phase, and *y*_Xe_ and *y*_Kr_ are their molar fractions in the bulk gas phase, here 0.2 and 0.8. Each point corresponds to a structure. The majority of the data points have selectivities between 1 and 10. As we are interested in the outstanding structures, and for clearer visibility, we masked this range of selectivities with a pink band. The size and *y*-positions of the structure points on the right of this band (*i.e.* very xenon-selective) correspond to their xenon uptakes. On the left side of the band, where structures are krypton-selective, the points' size and *y*-positions correspond to their krypton uptakes. The colours correspond to the data points cluster, indicated in Fig. 10e and f. For such separations, the ideal structure should be highly selective towards xenon whilst showing high xenon uptake. The highlighted area in red boxes is zoomed in Fig. 10c and d. The snapshots of some of the best-performing structures are also presented. Interesting structures are enclosed in orange boxes. For both MOCs and OCs, the cluster colours in Fig. 10c and d reveal families of structures with similar xenon uptakes and a range of selectivities (class 13 for MOCs and class 4 for OCs). Visual inspection of these structures reveals they are all CC3-type of structures. Fig. 10e and f present the boxplots of the different clusters, for selectivities over 10. The jittered points behind the boxplots indicate the number of data points involved. The markers represent the minimum, first quartile, median, third quartile, and maximum values, respectively, while the red dots indicate the average value in each boxplot. Outliers are shown with additional grey dots. For structures in this range of selectivities, MOC class 13 and OC class 4 indeed stand out as families with high values of selectivity. An image of a CC3-type of structure is given in Fig. 10e.

**Fig. 11:**
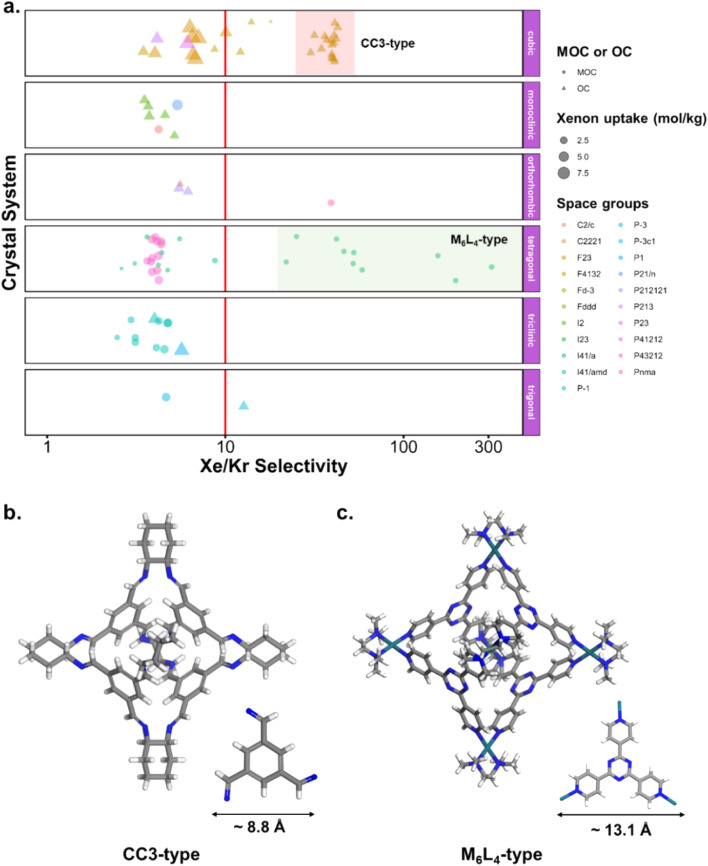
(a) Crystal systems of tetrahedral cages and their Xe/Kr selectivity. (b) Example of organic tetrahedral cage. (c) Example of metal–organic tetrahedral cage. In (a), each point corresponds to a structure. Its color corresponds to its space group, its shape to its classification as metal–organic cage (MOC) or organic cage (OC) and its size to its xenon uptake. The points are organised into different rows according to their crystal systems. The points are jittered in the *y*-axis for easier visualisation. The vertical red line indicates a selectivity of 10. The CC3-type structures are highlighted in orange. The M_6_L_4_-type structures are highlighted in green. One additional M_6_L_4_-type structure with a selectivity of 537 is not shown for clearer visualisation (CSD refcode: AJENIO^83^).

Fig. 10c and d revealed some of the best-performing structures, such as SISMUC and CIXBIX, both rings. Fig. 10e and f however show the statistical behaviour of the different classes of cages. While CC3 was not predicted to be the best-performing structure, its family of structures span a range of selectivity for a similar xenon uptake. The variability of the selectivity could be an artefact due to the very low uptake of krypton (close to 0), thus causing a large variance. However, it is also important to note that the classification here was only applied to the cage itself, and does not reflect the extrinsic pore shapes. Similar cages can pack differently, causing more or less efficiency in their selectivity. Because TDA cannot be applied directly to periodic systems, we looked at the crystal systems in which these structures crystallise. We gathered all tetrahedral cages and extracted their crystal system and space group information from the CSD. The results are shown in Fig. 11a. Each point corresponds to a structure. Its shape indicates whether it is a MOC or an OC and its colour its space group. The structures are organised into rows, each of which corresponds to their crystal system. The *x*-axis gives the xenon/krypton selectivity. The red line indicates the previously chosen threshold of selectivity equal to 10. Structures on the left side of the red line tend to have lower selectivity and higher xenon uptakes, whereas structures on the right side have higher selectivities and lower xenon uptakes. Interestingly, structures with higher selectivities that crystallise in cubic systems are also organic, while structures with higher selectivities that crystallise in tetragonal systems are also metal–organic. These two types of structures have distinct features: most of the organic structures with higher selectivities gather around selectivity values of about 40 and uptakes of around 2.5 mol kg^−1^. These structures – highlighted in orange – correspond to CC3 cages obtained under different conditions from the Cooper group.^6,80–82^ A typical CC3 structure is shown in Fig. 11b. The metal–organic structures, however, span a range of selectivities at lower xenon uptakes of around 1.6 mol kg^−1^ and correspond to the same M_6_L_4_ structures (6 metal nodes and 4 ligands) synthesised under different conditions by the Fujita group.^83–90^ These structures are highlighted in green and Fig. 11c shows their typical structure. The high variance of the selectivities calculated for these M_6_L_4_ cages is likely due to flexibilities not accounted for in the GCMC simulations. More details about the observed structural differences are provided in Section S6 of the ESI.†

## 4. Updating the datasets of structures

To offer the most up-to-date list of MOCs and OCs, and to demonstrate how these datasets can easily be amended with new structures and structures that were not taken into account, we have applied the same methods outlined here to (i) *ca.* 50 000 new metal–organic molecules and (ii) *ca.* 60 000 new organic molecules added between November 2019 and March 2022, as well as (iii) 76 cages found while targeting carboxylate-based cages.

To reduce the search space for MOCs and OCs, we first used ConQuest to look for any structures added between these two dates with the filters *3D coordinates determined*, *not polymeric* and *only organometallics* or *only organics*, respectively. We obtained a list of 51 923 structures for MOCs and 64 630 for OCs. After running our first script to remove fully linear structures, we were left with 50 304 potential MOC candidates and 62 702 OC candidates. We then obtained the TDA landscapes and performed the hierarchical clustering, after which we visually inspected the clusters obtained. 391 new MOCs and 814 new OCs were found using this method.

**Fig. 12:**
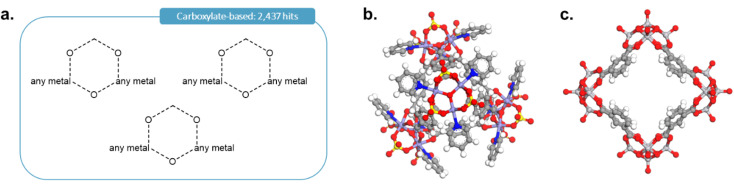
(a) Quick ‘must-have’ criteria drawn in ConQuest for carboxylate-based cages. The dotted lines refer to ‘Any’ type of bonds. When atoms are not explicitly indicated, they correspond to C atoms. Examples of targeted carboxylate-based cages: (b) CSD refcode: JANYUT,^91^ (c) CSD refcode: EXAMIB.^92^

As it was rightly pointed out to us, carboxylate-based cages form an important type of MOCs. To add the corresponding cages, we first performed the query shown in Fig. 12a in ConQuest, with the filters *3D coordinates determined*, *not polymeric* and *only organometallics*. This search targeted structures such as those shown in Fig. 12b and c and returned 2437 structures. After obtaining the TDA landscapes and performing the hierarchical clustering, one class of 76 structures stood out as carboxylate-based cages and rings.

We have thus updated the list of MOCs and OCs to contain 1839 and 7736 structures, respectively. The lists of MOCs and OCs determined in 2019 and these latest lists can all be found on our Github.

## 5. Conclusion

We presented the use of topological data analysis for the identification of cages in the CSD. In addition, we demonstrated the usefulness of hierarchical clustering in the unsupervised classification of cages, as well as in visualising the structures' similarity. Using these methods, we successfully obtained the first MOC dataset and an OC dataset which expands the OC space previously known by seven-fold. Whilst the presented procedure is more complex to integrate into the CSD for automatic updates, we suggest applying random forest on persistent homology landscapes to determine whether a new structure is a cage. We illustrated the information we obtained with a xenon/krypton separation simulation. We confirmed the high performance of the previously identified CC3 cages, whilst also identifying high-performance rings. More interestingly, we found the metal–organic equivalents to CC3 (M_6_L_4_) and compared their respective selectivities.

While the computational field of organic porous cages is growing fast, this is – to the best of our knowledge – the first extensive search of OCs in the CSD. A significant amount of work on the classification and prediction of OCs has already been produced, albeit relying on a cage-specific topology nomenclature – different from the mathematical concept of topology used in this work. Of the predicted 20 most common topologies defined by Santolini *et al.*,^93^ 12 have been experimentally reported. Greenaway *et al.* took a step further by creating a hybrid computational-experimental high-throughput workflow where conventional virtual HTS was combined with robotic synthesis to discover new cages.^25^ Of the 78 precursor combinations chosen, 33 cages were eventually synthesised and one previously unknown topology was discovered. Feeding and mapping the OCs dataset obtained in this work to the latent cage space derived by Sturluson *et al.* and to known cage topologies would bring invaluable additional insight to the regions that have already been explored and to possible directions for the discovery of new cages. Extending the same cage-specific topology definitions and mappings to MOCs could not only accelerate the computational discovery of MOCs, but also provide a clear research framework early on in the development of the field.

## Data availability

All the scripts used and the datasets obtained are available at: https://github.com/ayl23/TDA_cages.

## Author contributions

A. L. designed, performed and wrote the work presented in this paper. R. B.-P. and D. F.-J. supervised the work.

## Conflicts of interest

There are no conflicts to declare.

## Supplementary Material

SC-013-D2SC03171J-s001
