# Supplementary material for: Identifying porous cage subsets in the Cambridge Structural Database using topological data analysis
Source: Chem Sci. 2022 Oct 31;13(45):13507–23. doi: 10.1039/d2sc03171j (PMC9682994; doi:10.1039/d2sc03171j)
Supplement: SC-013-D2SC03171J-s001 [file SC-013-D2SC03171J-s001.pdf]

Electronic Supplementary Material (ESI) for Chemical Science.
This journal is © The Royal Society of Chemistry 2022

Supplementary Information for:

Identifying cages in the CSD using topological data analysis

Aurelia Li, Rocio Bueno-Perez, David Fairen-Jimenez
The Adsorption & Advanced Materials Laboratory (A^2ML), Department of Chemical Engineering & Biotechnology, University of Cambridge, Philippa Fawcett Drive, Cambridge CB3 0AS, UK

CONTENTS

S1 Persistent homology 2

S2 Descriptions of the MOC groups 4

S3 Examples of OCs identified 7

S4 Additional ConQuest queries used for reducing the search space of OCs in the CSD 9

S5 GCMC simulations 26

S6 CC3 vs M6L4 27

References 30

S1 Persistent homology

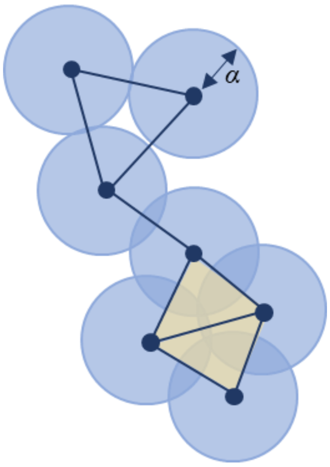

Figure S1 Vietoris-Rips filtration: a set of points where the distance between two points is less or equal than alpha.

Bottleneck distance:

A persistent diagram P is a set of points:

$$P = \{(b_i, d_i), d_i > b_i \geq 0, i = 1..n\}$$

Given two persistent diagrams P and Q, we want to determine their similarity. For this, we want to match points from P with points from Q and calculate their distance. However, P and Q do not necessarily have the same number of points, and some points in either diagram can be left out. Instead of defining a bijection between P and Q, we define a partial match. A partial match between P and Q is a bijection between a subset of P and a subset of Q. Let M be this partial match.

Given two points p and q of coordinates (b, d) and (b', d') respectively, we define their sup norm in $\mathbb{R}^2$ as:

$$||p - q||_\infty = \max\{|b - b'|, |d - d'|\}$$

We can, therefore, compute the sup norm of all the partially matched points. For unmatched points of coordinates (b, d), we take the sup norm to their closest point to the diagonal. The closest point has coordinates $½(b + d, b + d)$. For the unmatched points, we therefore take:

$$\left\|(b, d) - \frac{(b + d, b + d)}{2}\right\|_\infty = \frac{\mathrm{b - d}}{2}$$

Given P, Q, M and the defined sup norm, we define the cost of M as:

$$C(M) = \max\{\sup_i ||p_i - M(p_i)||_\infty,$$
$$\sup_i \left\{\frac{b_i - d_i}{2}, b_i \text{ and } d_i \text{ unmatched in } P\right\}, \sup_i \left\{\frac{b'_i - d'_i}{2}, b'_i \text{ and } d'_i \text{ unmatched in } Q\right\}\}$$

Finally, the bottleneck distance d_b between P and Q is defined as the cost of the most efficient partial match:

$$d_b(P,Q) = \min_{M:P\to Q} C(M)$$

S2 Descriptions of the MOC groups

Imidazole-based cages

The imidazole-based cages describe structures where the metal atoms are connected to the organic ligands via at least four nitrogen atoms, two of which should be part of an imidazole. As most targeted cages have at least four metal atoms, four identical units of such atoms connected to an imidazole are repeated. **Figure S3** gives three examples of structures obtained with this query. Note the variety of shapes: EHIHIN[1] is a tetrahedral cage, LAVMOM[2] has the shape of a funnel, and ZULJAT[3] is a helicate. 1,878 hits were obtained from this search.

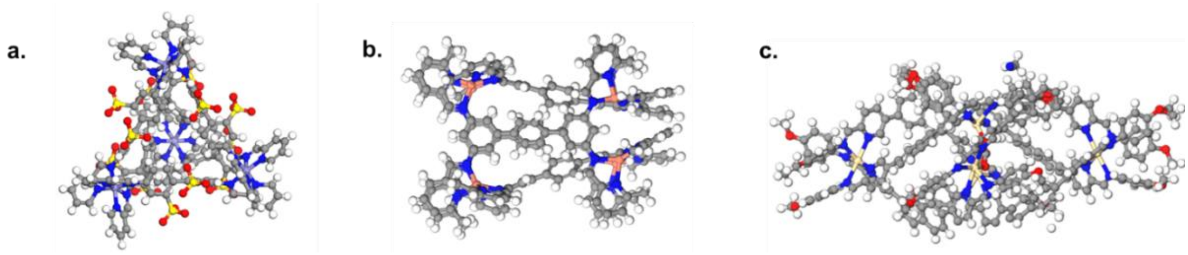

Figure S2 Examples of structures obtained with the imidazole-based query. CSD refcodes: **a.** EHIHIN,[1] **b.** LAVMOM[2] and **c.** ZULJAT.[3]

Pyridine-based cages

The pyridine-based query describes structures where the metal atoms are connected to four pyridine compounds, each of which is then connected to a carbon atom. In addition, each entry should have at least three metal atoms. The green dashed line in **Figure 5** separates the queries above and below, meaning only one of these pyridine units is necessary. These two queries should be combined in ConQuest as an AND statement. **Figure S4**Figure shows two examples of structures targeted with this query. 116 hits were obtained from this search.

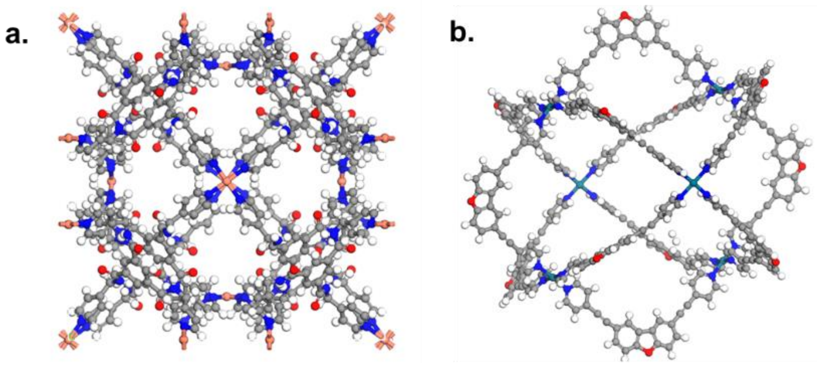

Figure S3 Examples of structures targeted with the pyridine-based cages query. CSD refcodes: **a.** CIYWOX[4] and **b.** COWBIA.[5]

Banana-shaped cages

The term 'banana' was coined by Han et al. to describe the shape of the ligands, and not actually the overall cage. For the sake of simplicity, we will refer to these structures as banana-shaped. An example of such a cage is shown in **Figure S5**Figure **a.** Other non-

banana-shaped cages can also be found with this query; an example of a spherical cage is given in **Figure S5**Figure **b**. 379 hits were obtained with this search.

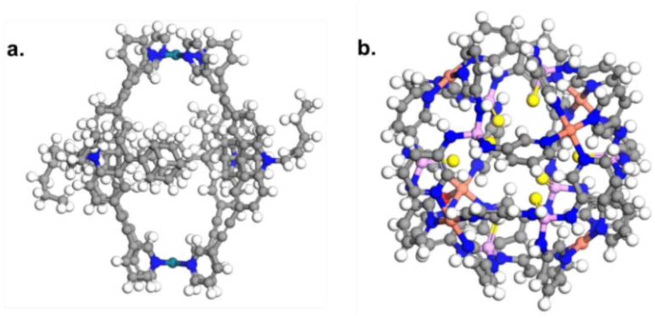

Figure S4 Examples of structures targeted by the banana-shaped query. CSD refcodes: **a.** ALEPEO,[6] **b.** AGEMAD.[7]

Bis(imino)pyridyl-based cages

The bis(imino)pyridyl-derived query describes structures where the metal atoms are part of a group containing two imidazole units which share the metal-nitrogen bond, and a pyridine unit which shares a bond and a nitrogen atom with each of the imidazole units. **Figure S6** gives two examples of cages obtained with this query. Note that ZOKDEL[8] in **Figure S6b** is referred to by the original authors as a macrocycle. The presence of a hollow in the macrocycle means it qualifies as a cage, in the case of our definition. 192 hits were obtained with this search.

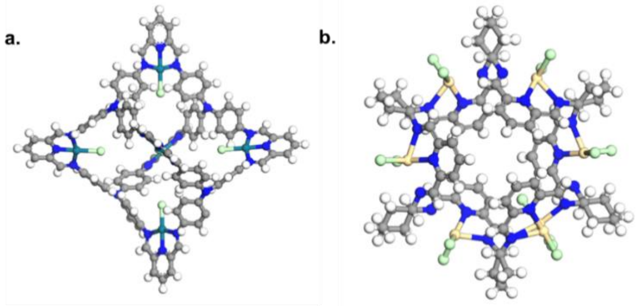

Figure S5 Examples of structures obtained with the bis(imino)pyridyl query. CSD refcodes: **a.** VOMGOW,[9] **b.** ZOKDEL.[8]

Dioxolane/dioxane-based cages

The dioxolane/dioxane-based query addresses the case of structures where the metal atoms are connected to either a 1,3-dioxolane or a 1,3-dioxane, as well as variants of these heterocycles where certain carbon atoms can be replaced with nitrogen atoms. **Figure S7** shows three examples of cages of different shapes – cylinder (ADODUS[10]), helicate (ANITAT[11]) and tetrahedral (BOBZUP[12]) – obtained with this query. 525 hits were obtained with this search.

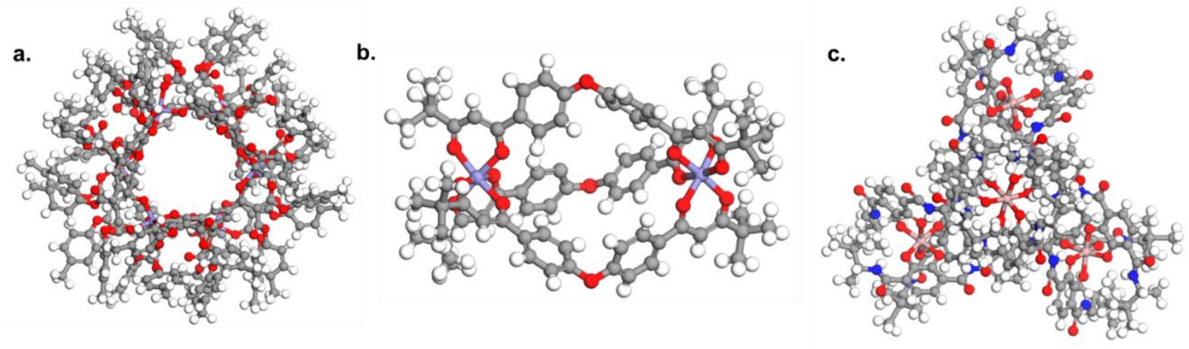

Figure S6 Examples of structures targeted by the dioxolane/dioxane-based query. CSD recodes: **a.** ADODUS,[10] **b.** ANITAT[11] and **c.** BOBZUP.[12]

Cyclotriveratrylene-derived cages

This query tackles specifically the emerging field of cyclotriveratrylene-derived coordination cages. As the essence of these cages lies in their organic ligand, the query consists in the description of the cylotriveratrylene ligand, accompanied by the presence of at least two metal atoms. **Figure S8** gives two examples of such cages with different shapes. These cages are prone to structures with multiple cavities. **Figure S8c** gives an example of such a structure, where two cages, each with two distinct pores, are linked via an organic ligand. 85 hits were obtained with this search.

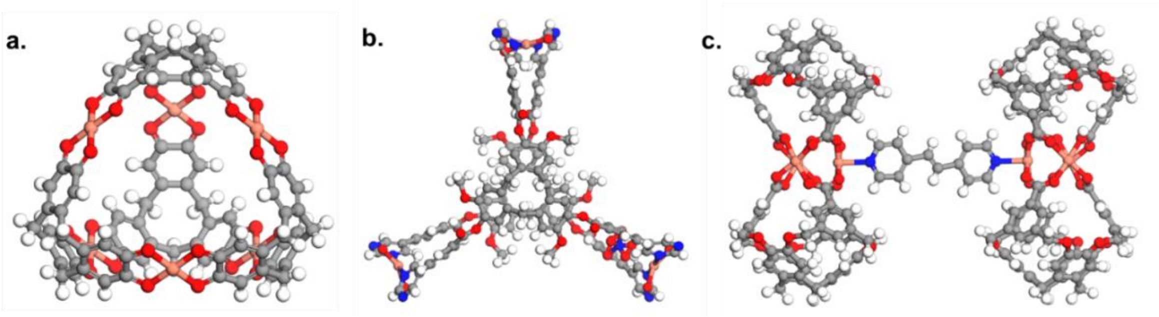

Figure S7 Example of cages obtained with the cyclotriveratrylene-derived query. CSD refcodes: **a.** ATOXIR,[13] **b.** UTADOJ[14] and **c.** EHEJAD.[15]

Large cages

Some cages are too large and do not have an assigned 2D chemical diagram, which means a substructure search in ConQuest will miss them. However, these structures have the word 'exceeded' in their textual description. This search returned 612 hits.

S3 Examples of OCs identified

Carbon-based cages

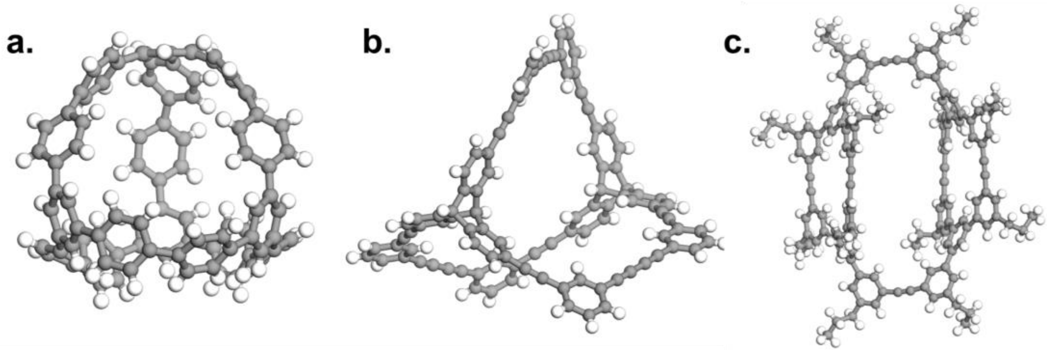

Figure S8 Examples of targeted carbon-based cages. CSD refcodes: **a.** CIMCIM,[16] **b.** LISTOX[17] and **c.** YOHXOK.[18]

Imine-based cages

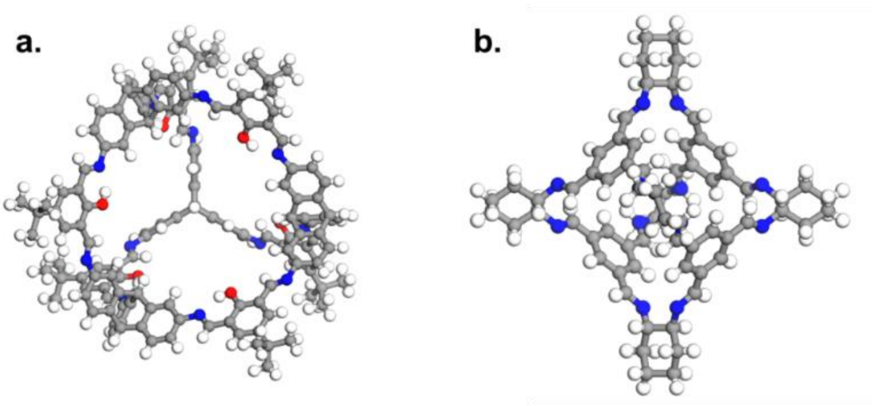

Figure S9 Examples of targeted imine-based cages. CSD refcodes: **a.** EKUKUR[19] and **b.** FOXLAG.[20]

Boronate-based cages

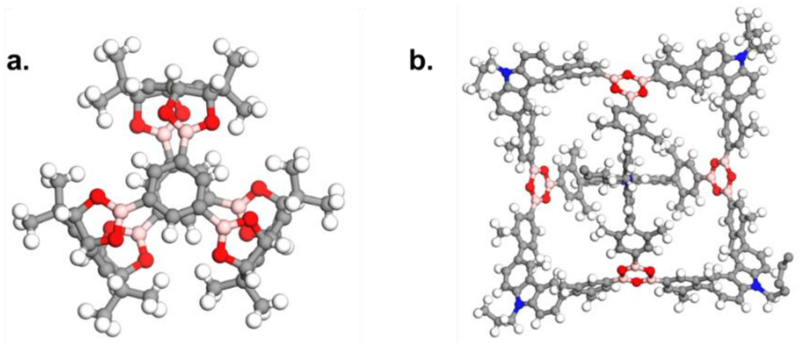

Figure S10 Examples of targeted boronate-based cages. CSD refcodes: **a.** AJOHUD[21] and **b.** YUKHOD.[22]

Oxygen-based cages

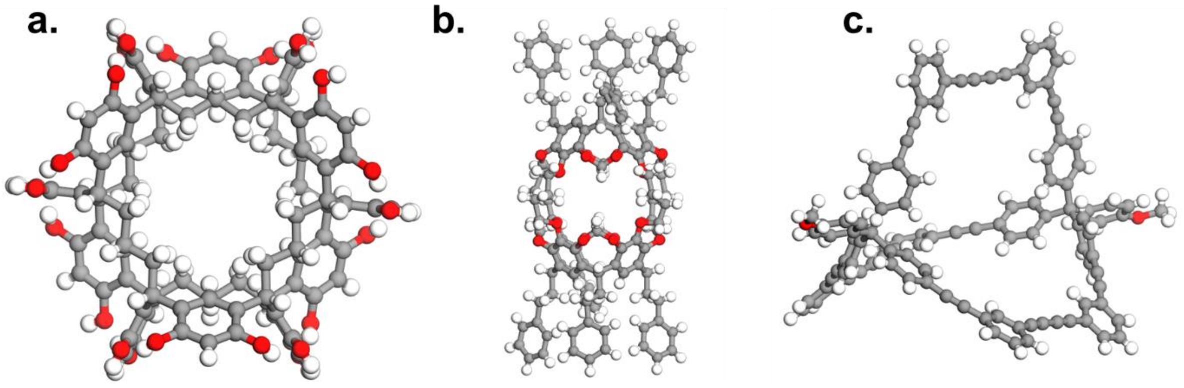

Figure S11 Examples of targeted oxygen-based cages. CSD refcodes: **a.** GUMCIB,[23] **b.** PAQFES[24] and **c.** REQXES.[25]

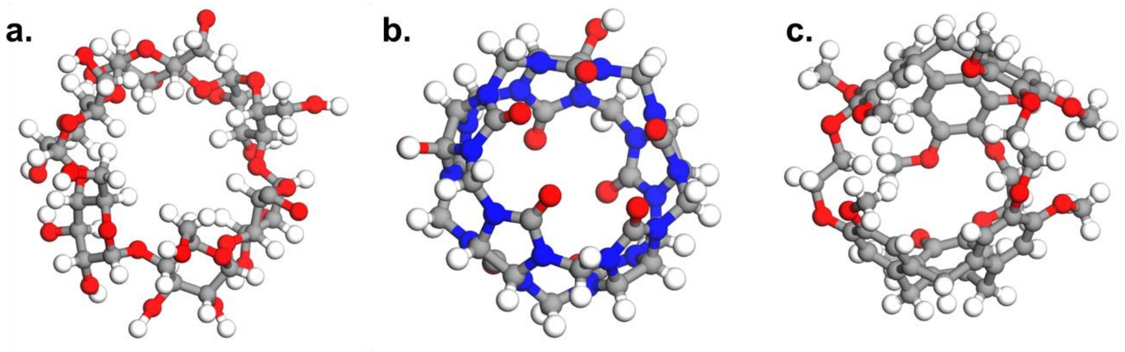

Figure S12 Examples of **a.** cyclodextrins, **b.** cucurbiturils and **c.** cryptophanes. CSD refcodes: **a.** ACDHBA,[26] **b**. AHUPOK,[27] **c.** XIHQAI.[28]

S4 Additional ConQuest queries used for reducing the search space of OCs in the CSD

The following queries for organic cages and rings were added to the general queries for OCs. Dotted lines correspond to ‘any’ type of bond. Superscript c means the corresponding atom should be cyclic. Superscript a means the corresponding atom should be acyclic. Sub-queries highlighted in a red box refer to ‘must not have’ criteria. ‘TN’ means the corresponding atom is attached to N other atoms only.

Table S1 Additional queries for organic cages.

Query	Number of hits
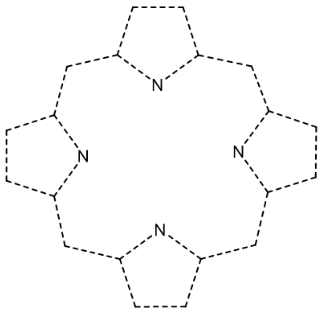 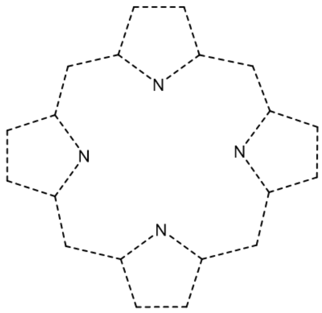	105
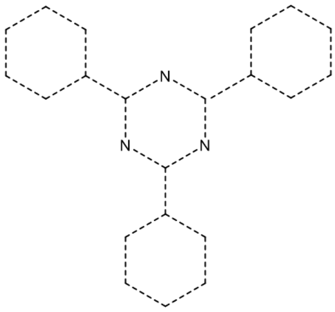 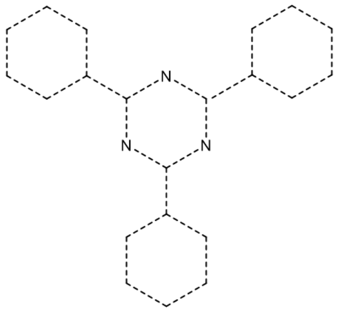	22
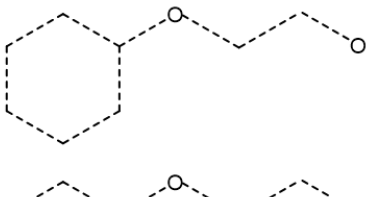 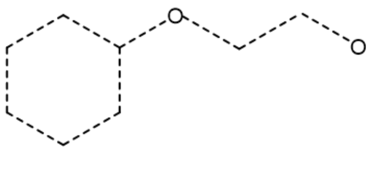	3670

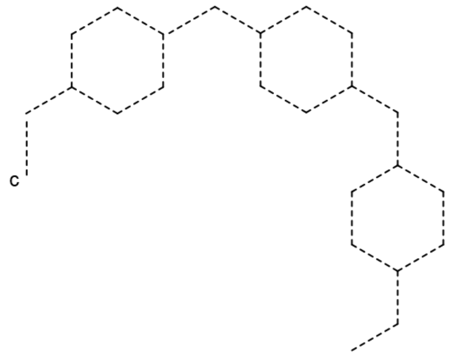

1567

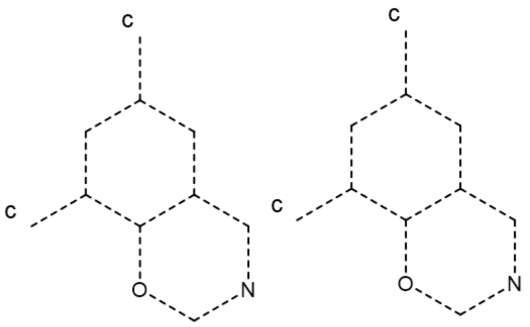

17

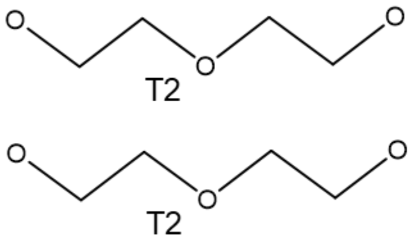

3793

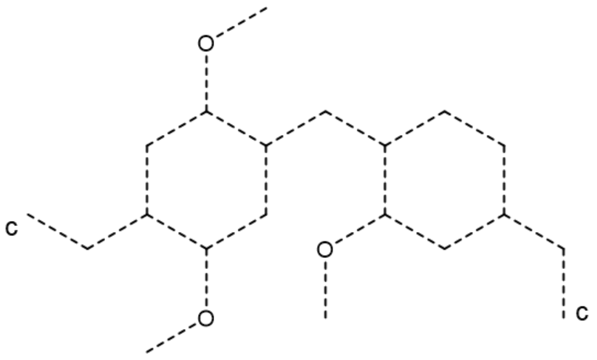

329

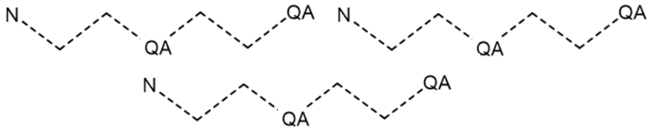

1867

QA = O or N

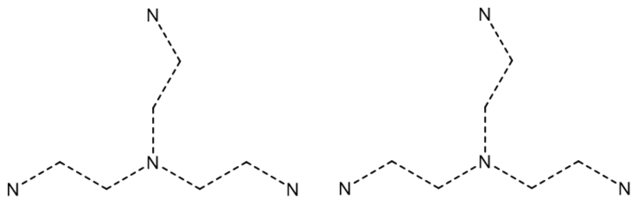

334

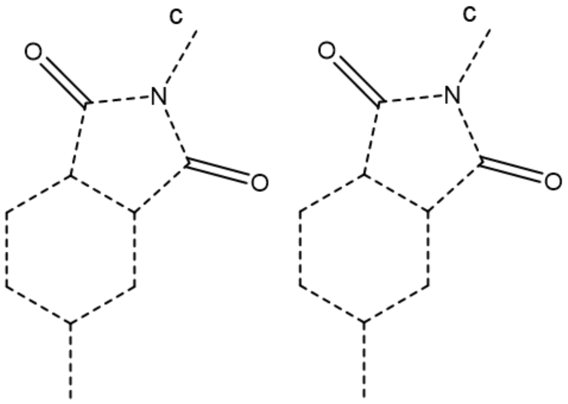

71

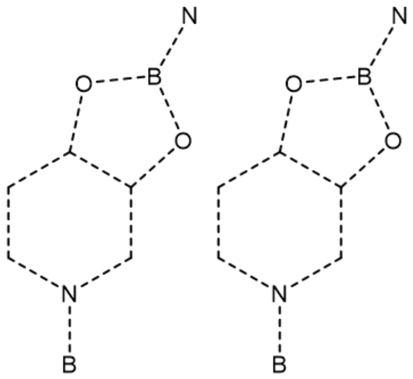

4

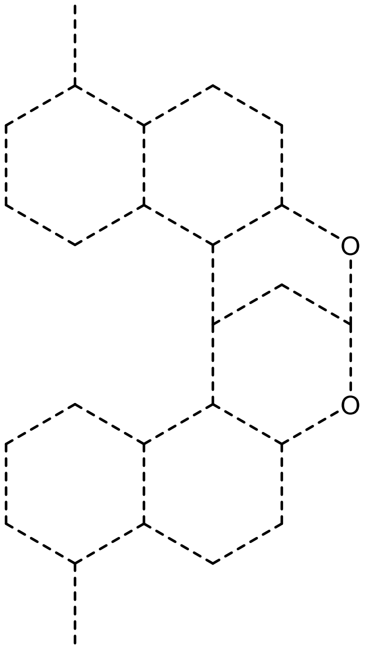

39

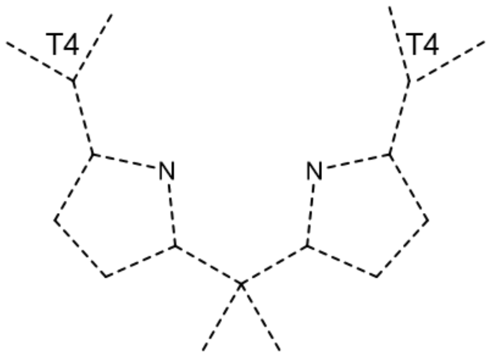

529

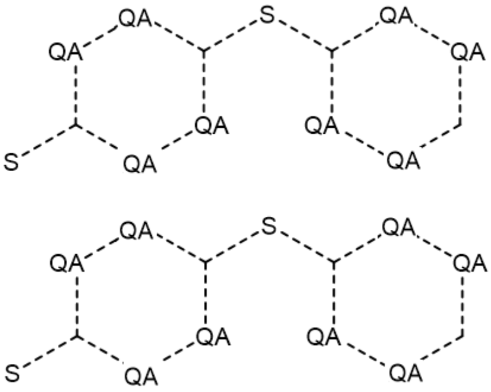

70

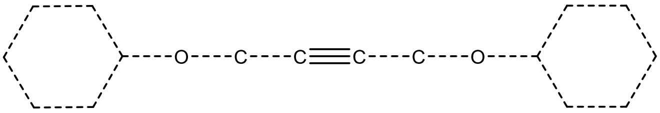

47

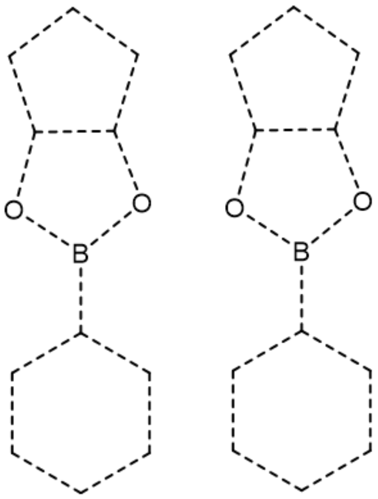

30

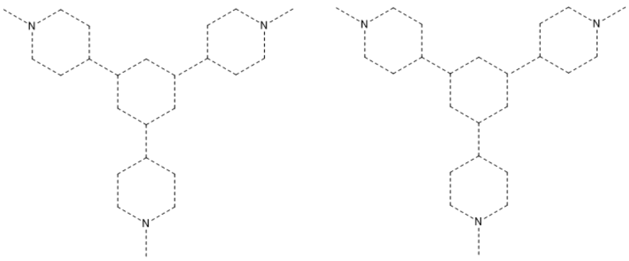

10

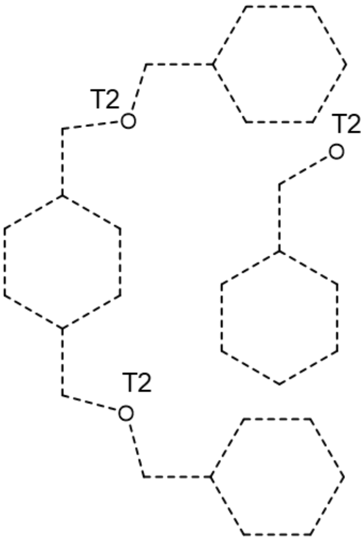

35

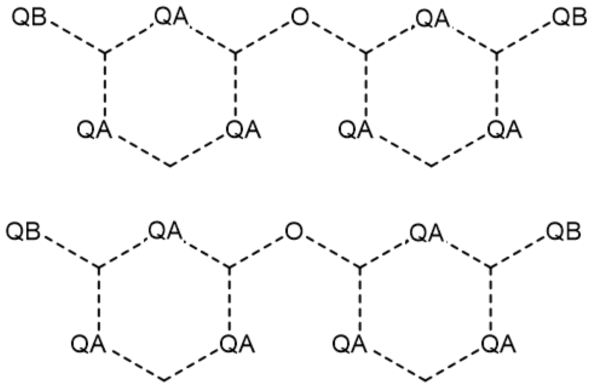

245

QA = C or N, QB = C or O

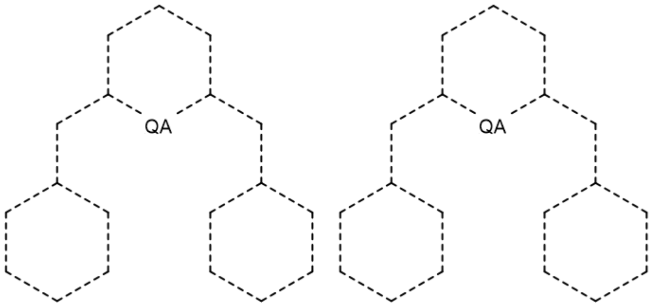

87

QA = C or N

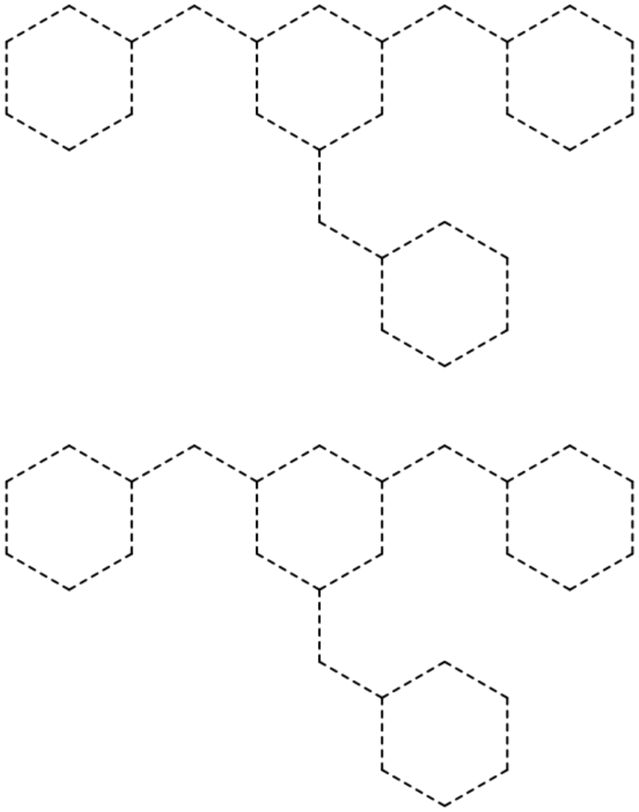

1494

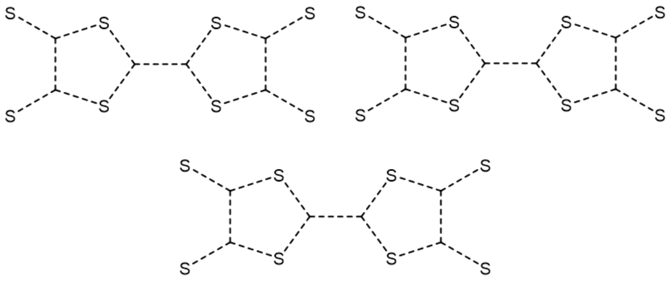

10

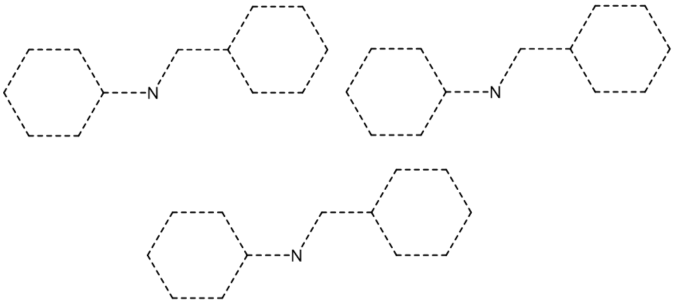

605

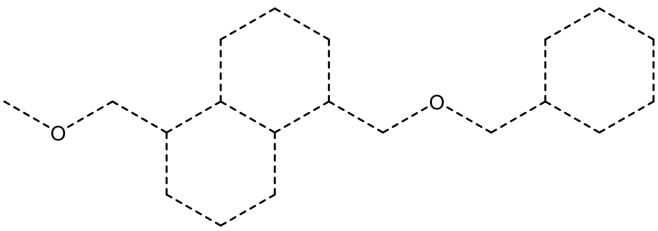

16

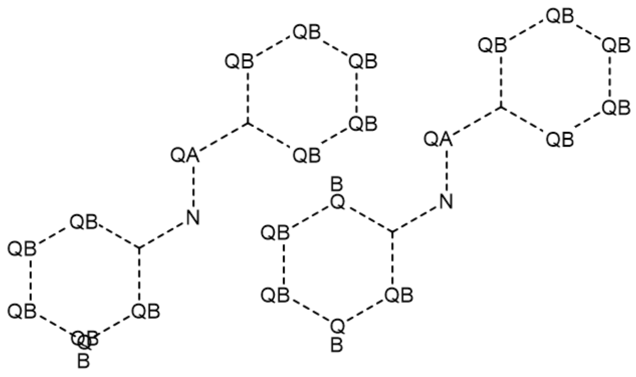

4036

QA = C or S, QB = C or N

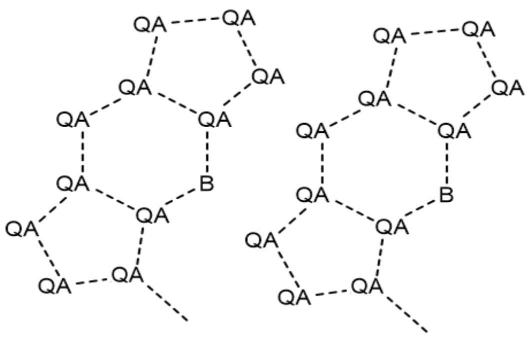

160

QA = C or N

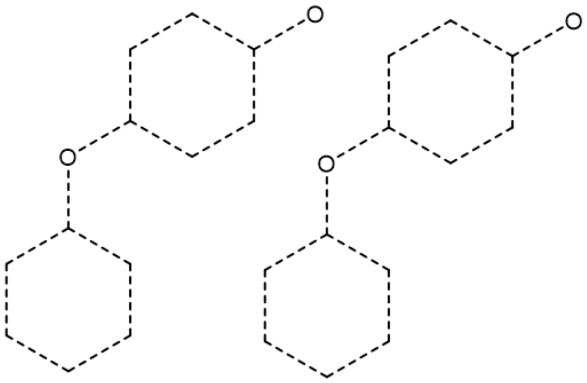

64

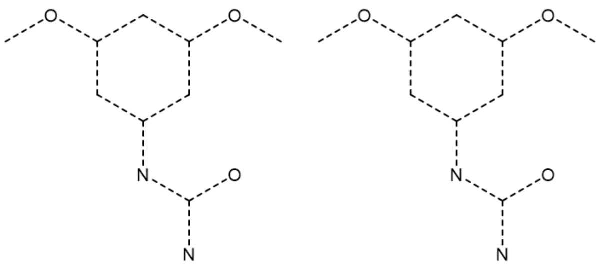

5

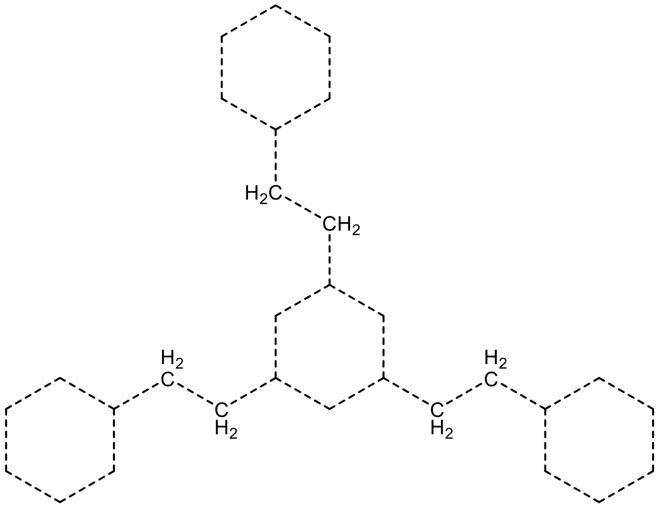

11

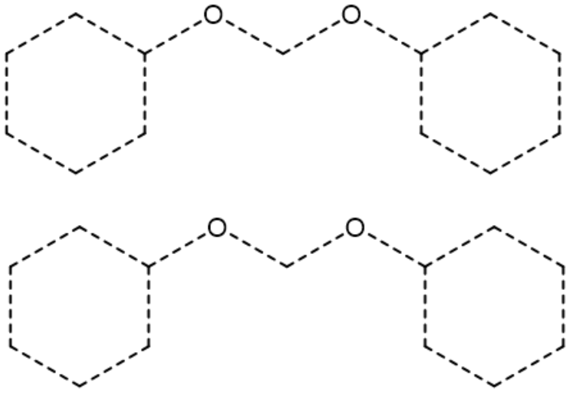

296

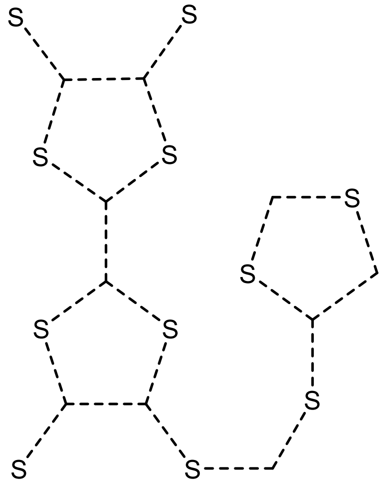

13

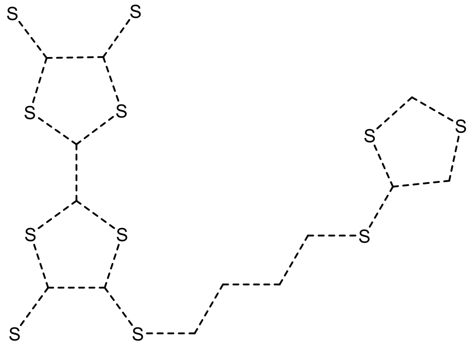

3

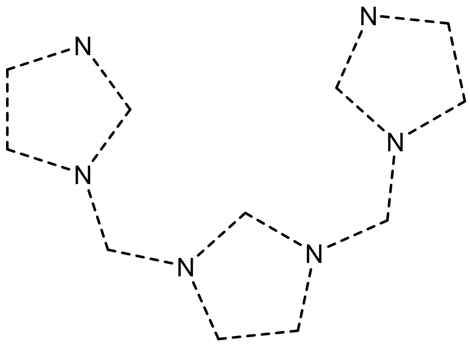

575

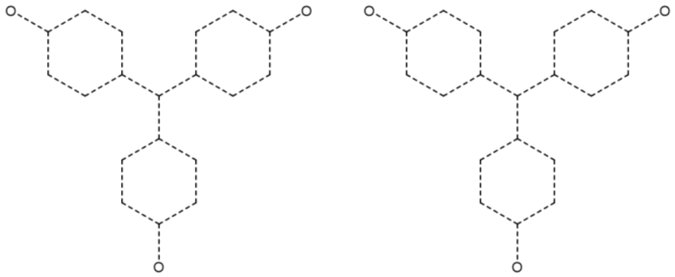

53

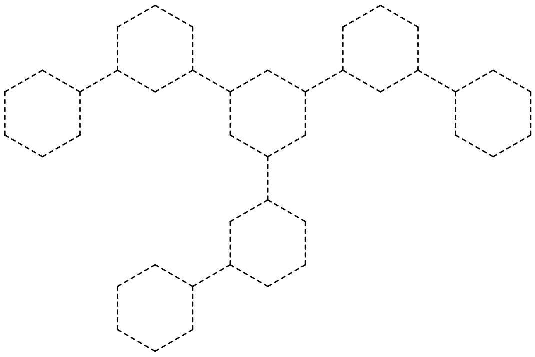

1561

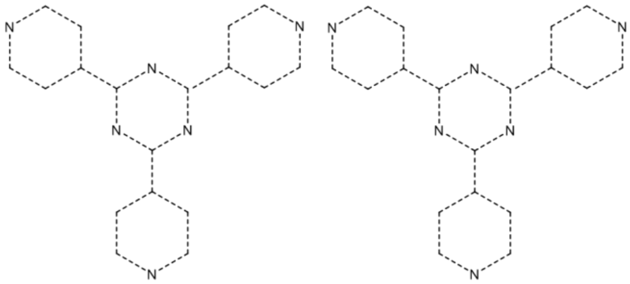

7

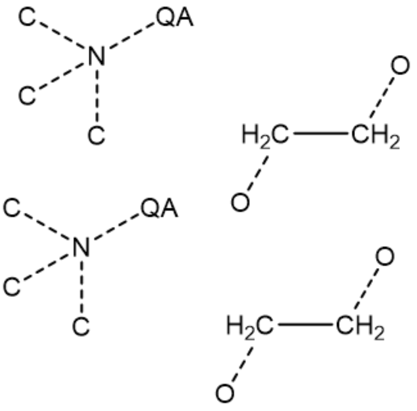

75

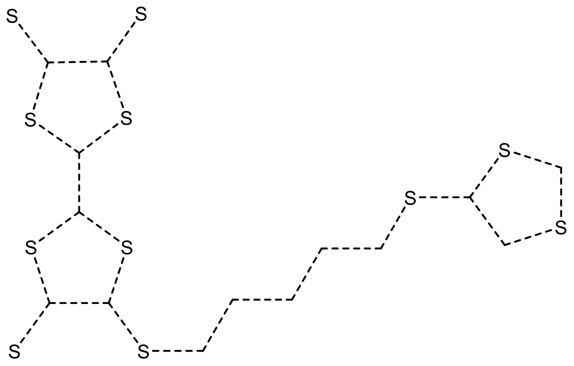

5

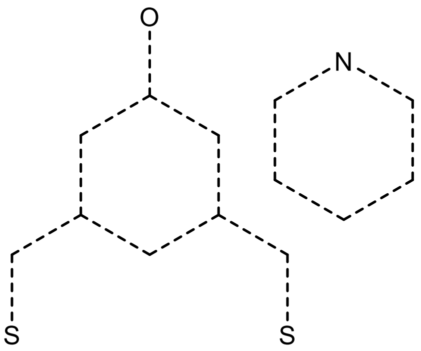

3

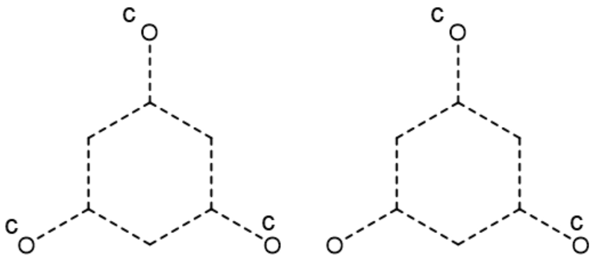

213

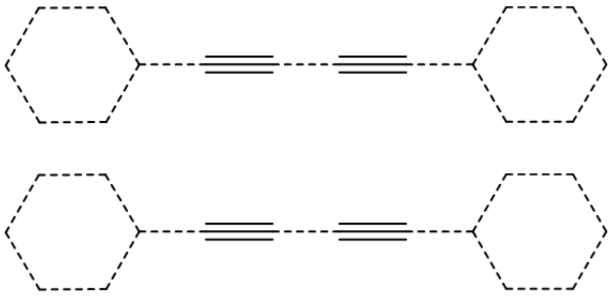

79

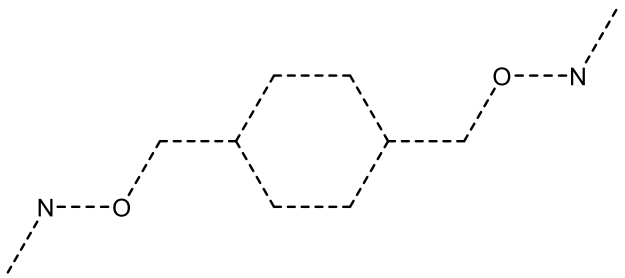

9

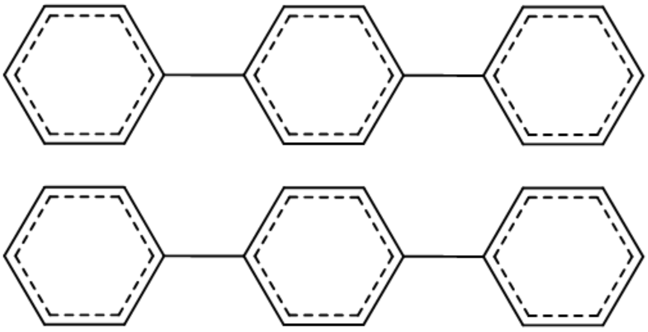

462

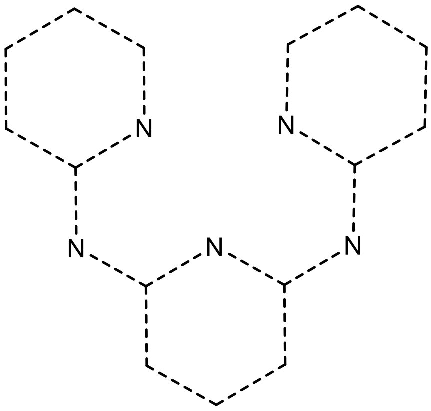

79

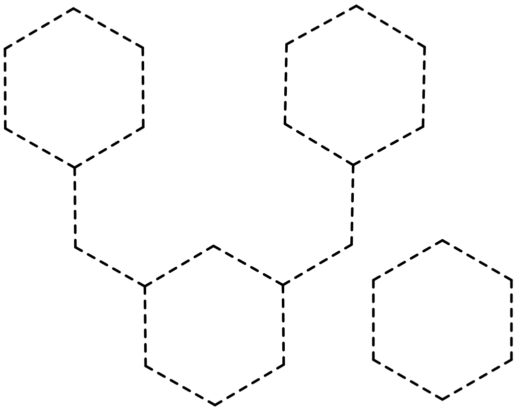

27

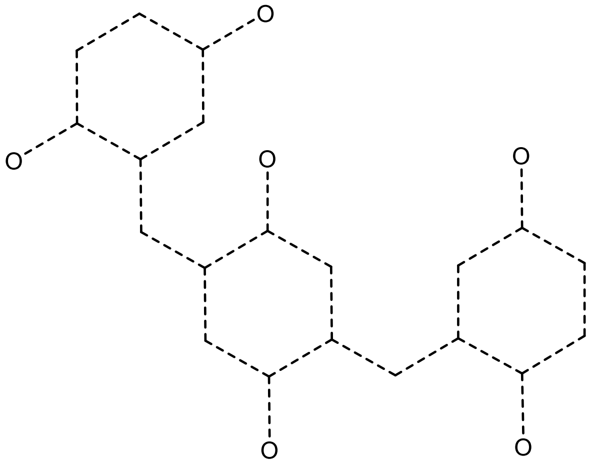

339

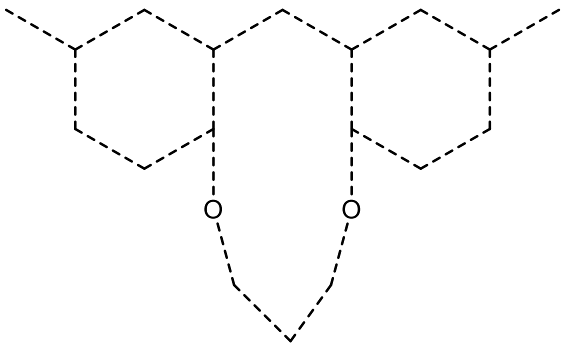

6

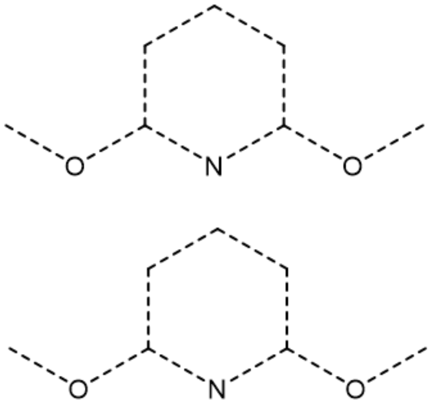

38

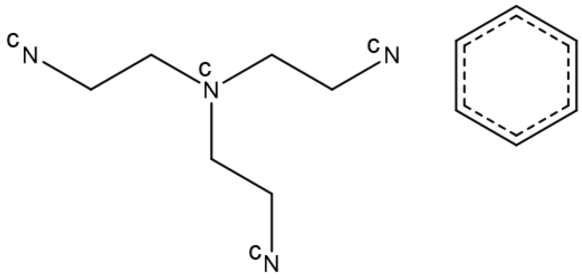

362

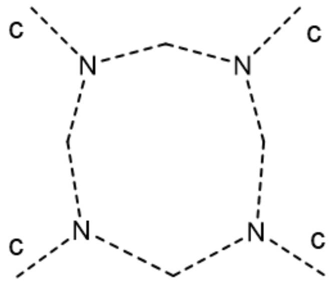

796

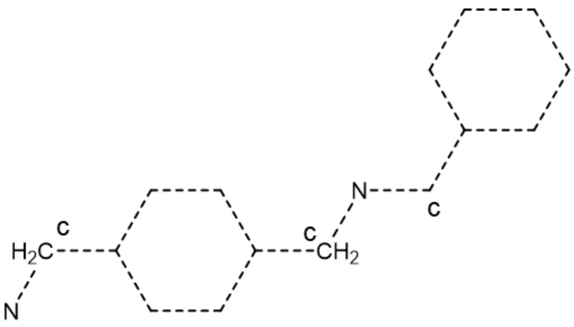

166

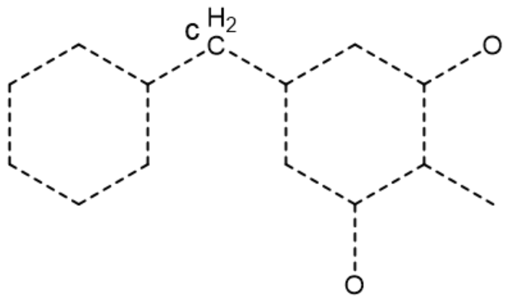

29

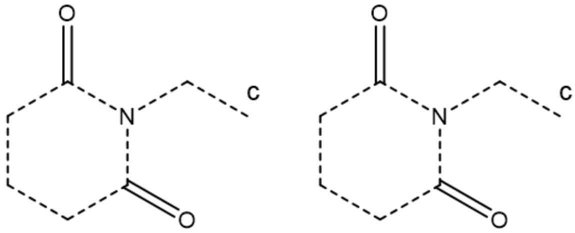

369

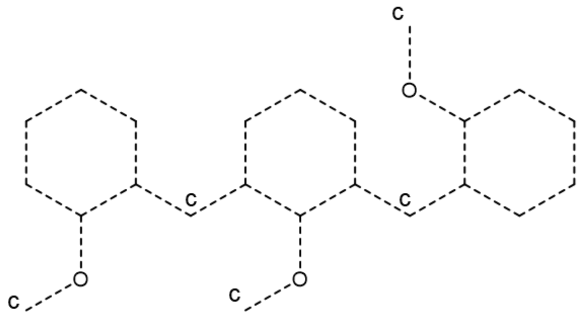

145

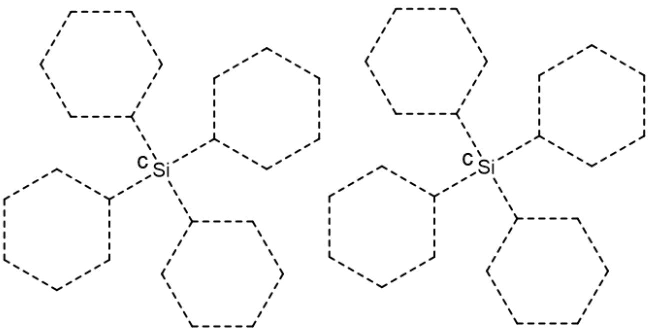

3

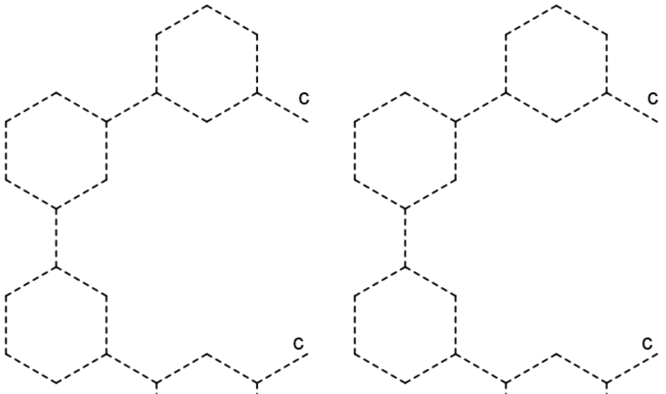

15

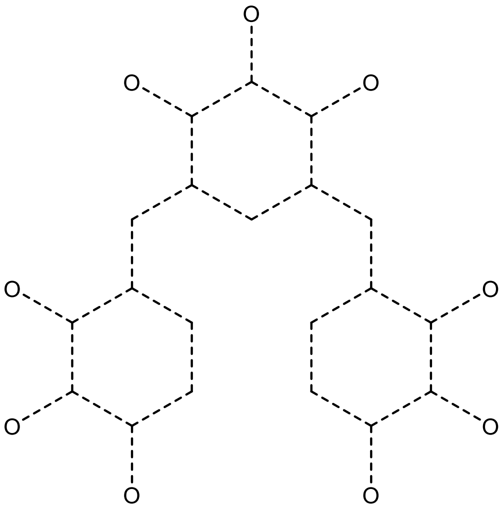

252

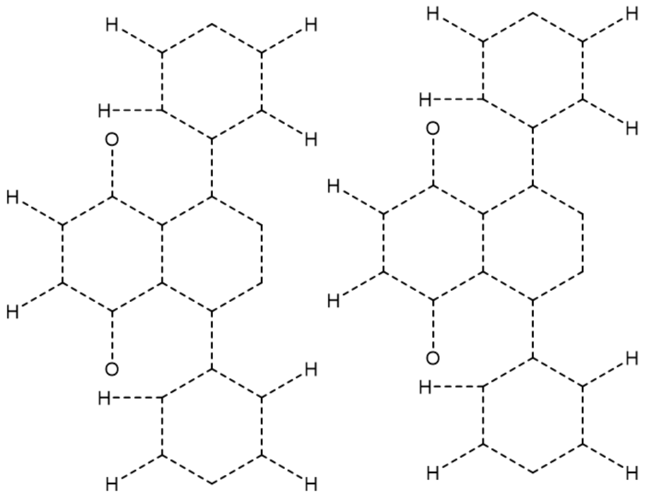

4

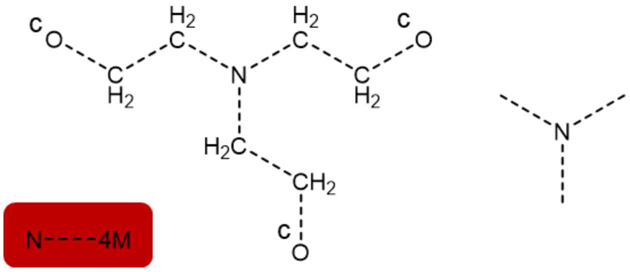

169

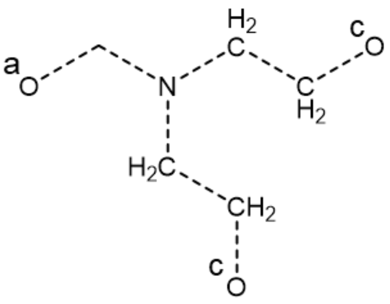

53

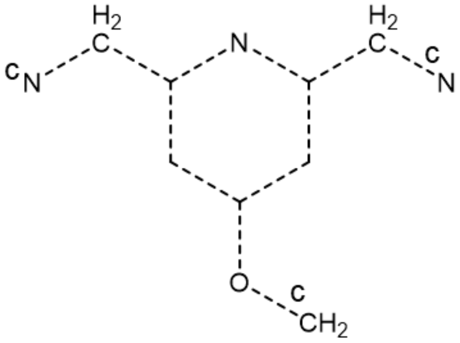

1

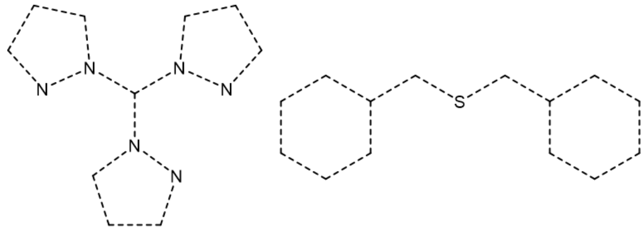

1

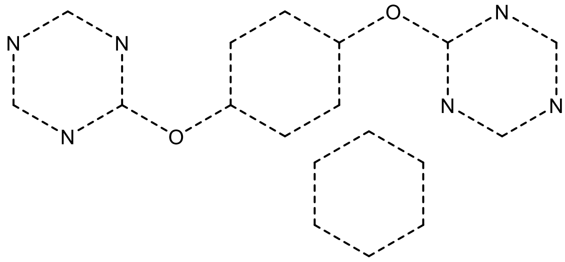

1

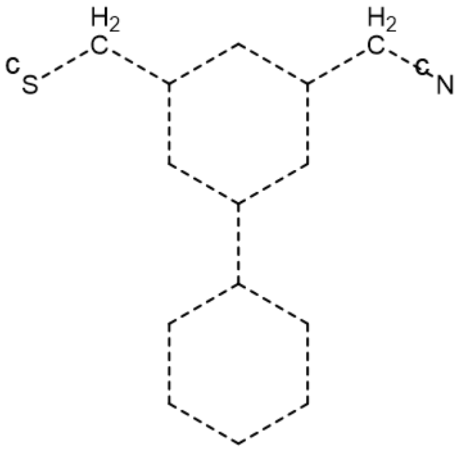

2

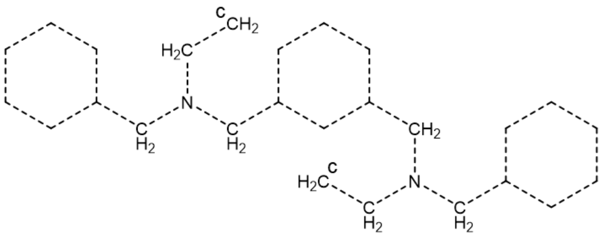

7

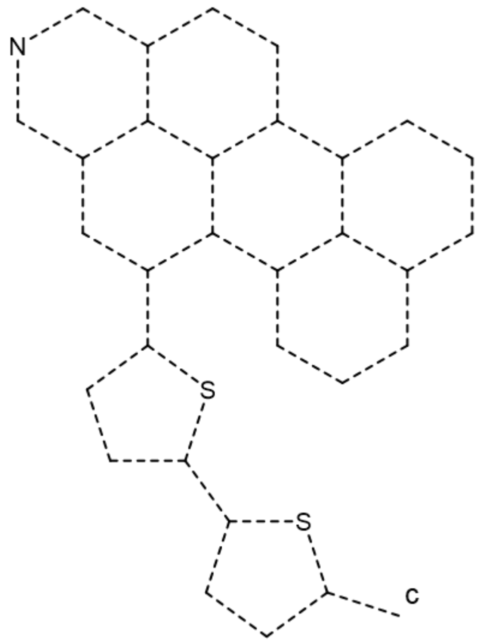

1

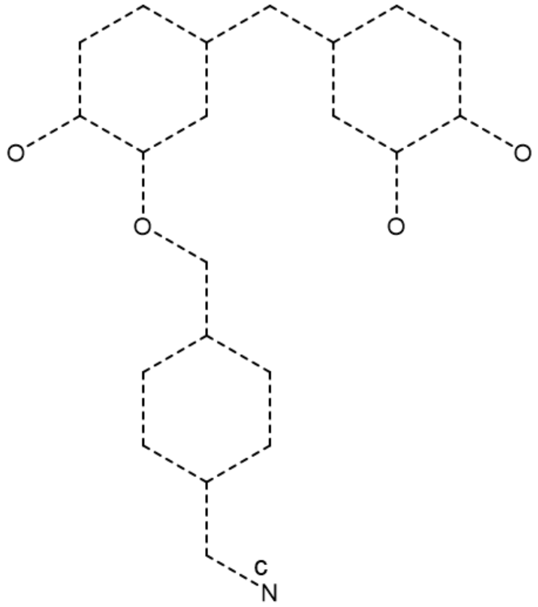

1

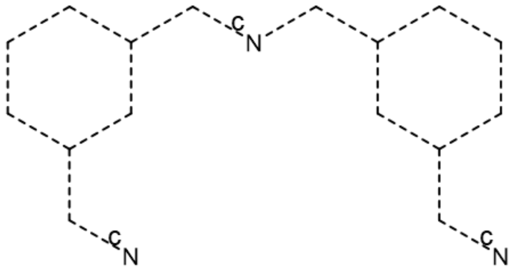

126

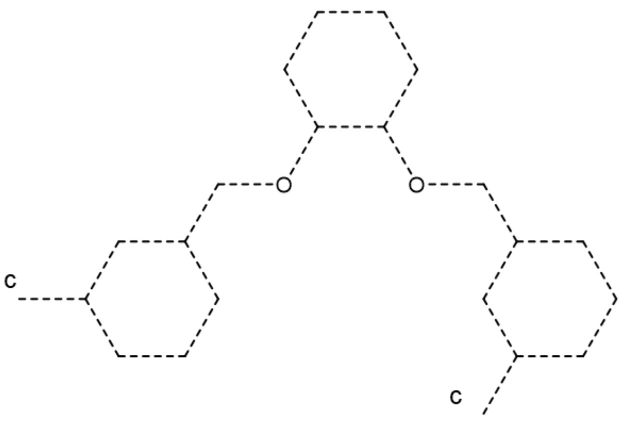

10

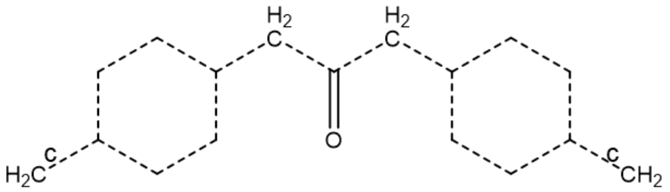

16

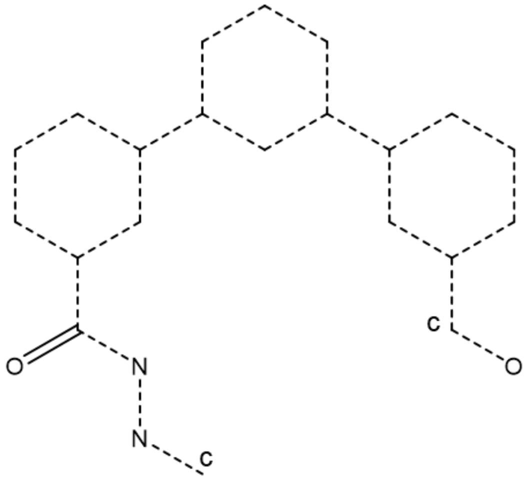

1

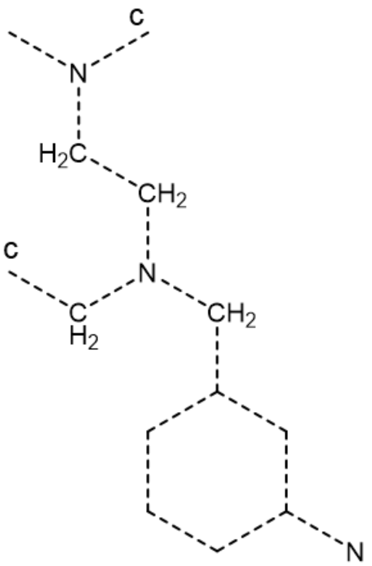

7

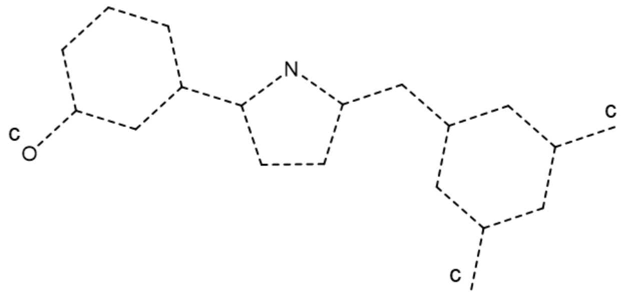

1

S5 GCMC simulations

We used the multi-purpose code RASPA to perform GCMC simulations of the said mixture in the selected MOCs and OCs.[29] We used an atomistic model of each clean structure where the atoms were kept fixed at their crystallographic positions. We used the standard Lennard-Jones (LJ) 12-6 potential to model the interactions between the framework and fluid atoms. The parameters for the framework atoms were obtained from Dreiding Force Field (DFF)[30] and, when not available, from the Universal Force Field (UFF).[31] The Lorentz-Berthelot mixing rules were employed to calculate fluid-solid LJ parameters, and LJ interactions beyond the cutoff value of 12.8 Å were neglected. The simulation box for each structure is defined so that the cell lengths are larger than twice the cutoff distance. 20,000 Monte Carlo cycles were performed, the first third of which were used for equilibration and the remaining steps for production. Monte Carlo moves consisted of insertions, deletions and displacements. In a cycle, N Monte Carlo moves were attempted, where N is defined as the maximum of 20 or the number of adsorbates in the simulation box. To calculate the gas-phase fugacity we used the Peng-Robinson equation of state.[32]

S6 CC3 vs M6L4

In this section, we attempt to explain the high variance observed for the same M_6L_4 cages observed in **Figure 11** (as opposed to the low variance demonstrated by the CC3 cages). For this, we visually compared two M_6L_4 structures: one at the relatively lower selectivity of 25 (CSD refcode: COPPAA[33]) and the structure with the highest selectivity (CSD refcode: AJENIO[34]). The two structures are presented in **Figure S13**. Although the individual cages share the same ligands, metal nodes and space groups, the size of the cells and the void fraction differ. Using the CCDC software Mercury of structure visualisation and analysis,[35] we computed the surface surrounding the porous areas in both structures. The result in **Figure S13** shows that the two surfaces differ significantly in shape. While a continuous channel runs through COPPAA from left to right, this channel is cut short in AJENIO. By comparing the two structures, we found that this difference in channel morphology is due to the difference in the bending of the organic ligands. To go from **Figure S13a** to **Figure S13b**, one can imagine pulling on the ligands at their centre in their perpendicular direction. This movement is indicated in **Figure S13a** by the yellow arrows. This difference in ligand bending possibly caused the observed differences in cell lengths, leading to an overall larger cell in the case of AJENIO, as well as larger pore volumes. These structural differences seem to have a large impact on the observed selectivities: a difference of 1 to 3% in cell lengths is related to a 33% difference in void fraction and one selectivity that is 21 times higher than the other. While the exact mechanism behind the difference in selectivity could be further investigated, the main take-away from this example is that slight differences in ligand bending lead to differences in the pores morphology that can have a significant impact on the calculated performance of the structures. These different bending angles could themselves be caused by different synthesis conditions, or could correspond to different states of a flexible structure.

Such high-impact structural variations were however not observed in the CC3-type structures. There are two possible reasons for this:

1. As shown in Error! Reference source not found.**b** and **c**, CC3 structures have shorter ligands which are therefore harder to bend.
2. CC3 structures crystallise in cubic systems, which provide more efficient packing and less leeway for structural variations. **Figure S14** shows the differences in packing in the two systems. This results in cages that are structurally extremely close, despite having been obtained under different conditions. The low structural variance in turns explains the observed low selectivity variance.

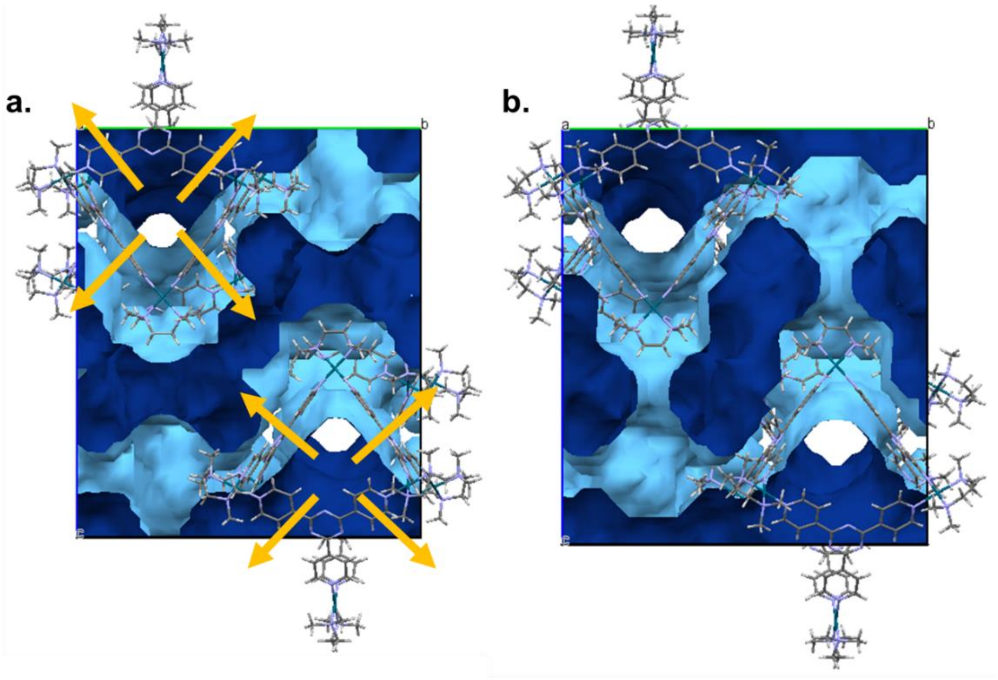

COPPAA
a = b = 26.216 Å
c = 31.195 Å
Cell volume = 21439.7 $Å^3$
Void fraction = 0.42
64 Xe molecules
10 Kr molecules
Xe/Kr selectivity = 25

AJENIO
a = b = 26.992 Å
c = 30.755 Å
Cell volume = 22407.1 $Å^3$
Void fraction = 0.56
67 Xe molecules
1 Kr molecule
Xe/Kr selectivity = 537

Figure S13 Comparison of two M_6L_4 structures with widely different Xe/Kr selectivity values: **a.** COPPAA and **b.** AJENIO. The blue surface maps out the porous areas, obtained in Mercury with a probe of radius 1.83 Å, corresponding to krypton's Van der Waals radius. The light blue corresponds to the outer surface and the dark blue to the inner surface. The yellow arrows in **a.** indicate the bending direction of the ligands to reach the cage morphology of AJENIO.

While we were able to shed some light on the spread of selectivity values observed for M_6L_4 cages, this case study revealed how sensitive simulations can be to slight structural differences among similar or identical structures obtained under different conditions, or captured in different flexibility states. These cases show the limit of assuming a host structure as rigid in molecular simulations, but also the distribution of different possible states for a given structure.

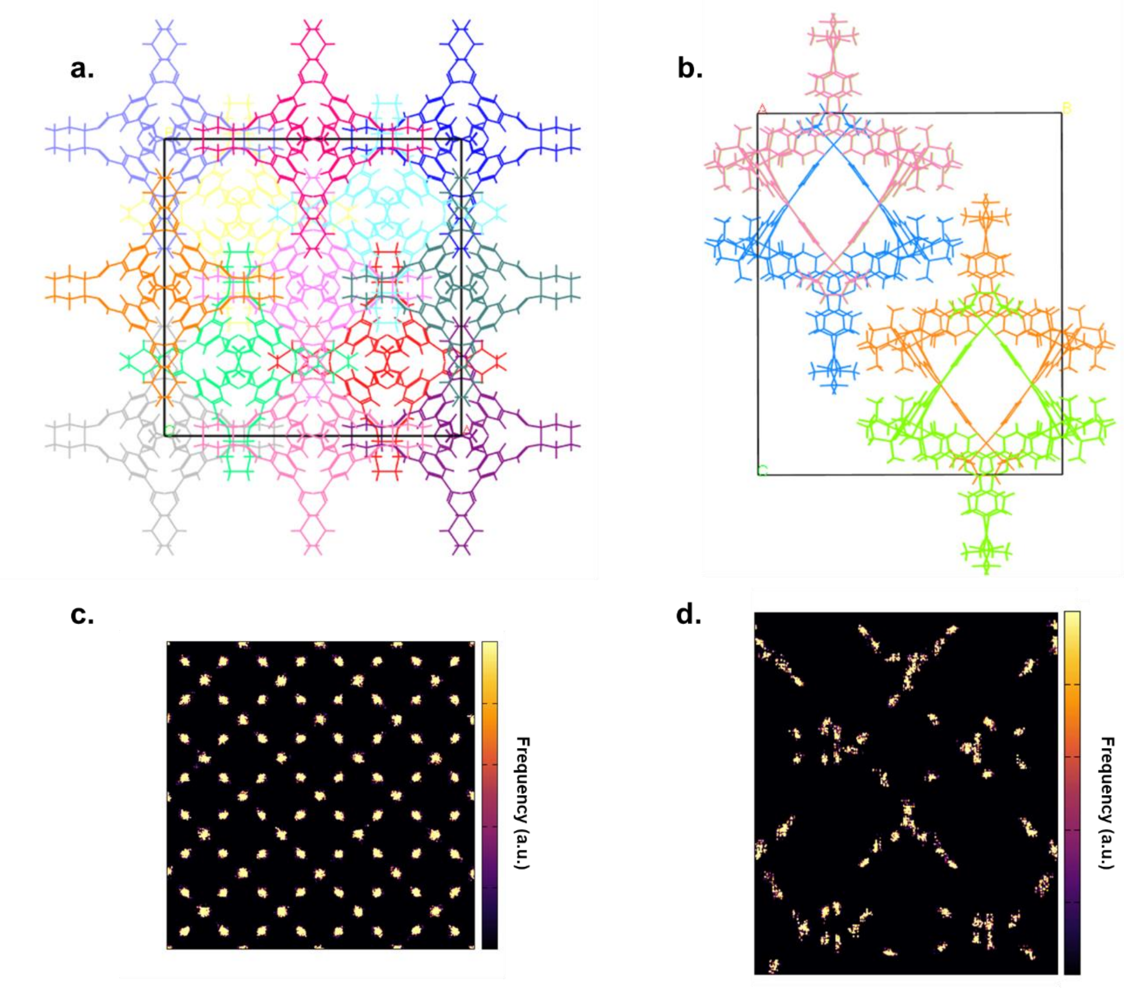

Figure S14 Differences in packing between CC3-type structures and M_6L_4-type structures. **a.** CC3 in its cubic system and **b.** COPPAA[33] in its tetragonal system. The cages are coloured for easier visualisation. The corresponding adsorption sites (obtained with SITES ANALYZER)[36] are shown in **c.** for CC3 and **d.** for COPPAA.

References

1. I. A. Riddell, M. M. J. Smulders, J. K. Clegg and J. R. Nitschke, *Chemical Communications*, 2011, **47**, 457-459.
2. W. Meng, J. K. Clegg and J. R. Nitschke, *Angewandte Chemie International Edition*, 2012, **51**, 1881-1884.
3. C. Browne, W. J. Ramsay, T. K. Ronson, J. Medley-Hallam and J. R. Nitschke, *Angewandte Chemie International Edition*, 2015, **54**, 11122-11127.
4. Y. Wang, T.-a. Okamura, W.-Y. Sun and N. Ueyama, *Crystal Growth & Design*, 2008, **8**, 802-804.
5. K. Suzuki, M. Tominaga, M. Kawano and M. Fujita, *Chemical Communications*, 2009, 1638-1640.
6. S. Löffler, J. Lübben, A. Wuttke, R. A. Mata, M. John, B. Dittrich and G. H. Clever, *Chemical Science*, 2016, **7**, 4676-4684.
7. A. Yadav, P. Kulkarni, B. Praveenkumar, A. Steiner and R. Boomishankar, *Chemistry – A European Journal*, 2018, **24**, 14639-14643.
8. R. Frydrych, K. Ślepokura, A. Bil and J. Gregoliński, *The Journal of Organic Chemistry*, 2019, **84**, 5695-5711.
9. R. Lavendomme, T. K. Ronson and J. R. Nitschke, *Journal of the American Chemical Society*, 2019, **141**, 12147-12158.
10. R. W. Saalfrank, H. Glaser, B. Demleitner, F. Hampel, M. M. Chowdhry, V. Schünemann, A. X. Trautwein, G. B. M. Vaughan, R. Yeh, A. V. Davis and K. N. Raymond, *Chemistry – A European Journal*, 2002, **8**, 493-497.
11. F. Li, J. K. Clegg, L. F. Lindoy, R. B. Macquart and G. V. Meehan, *Nature Communications*, 2011, **2**, 205.
12. C. Zhao, Q.-F. Sun, W. M. Hart-Cooper, A. G. DiPasquale, F. D. Toste, R. G. Bergman and K. N. Raymond, *Journal of the American Chemical Society*, 2013, **135**, 18802-18805.
13. B. F. Abrahams, B. A. Boughton, N. J. FitzGerald, J. L. Holmes and R. Robson, *Chemical Communications*, 2011, **47**, 7404-7406.
14. J. J. Henkelis, T. K. Ronson, L. P. Harding and M. J. Hardie, *Chemical Communications*, 2011, **47**, 6560-6562.
15. T. K. Ronson, H. Nowell, A. Westcott and M. J. Hardie, *Chemical Communications*, 2011, **47**, 176-178.
16. E. Kayahara, T. Iwamoto, H. Takaya, T. Suzuki, M. Fujitsuka, T. Majima, N. Yasuda, N. Matsuyama, S. Seki and S. Yamago, *Nature Communications*, 2013, **4**, 2694.
17. C. Zhang and C.-F. Chen, *The Journal of Organic Chemistry*, 2007, **72**, 9339-9341.
18. Q. Wang, C. Zhang, B. C. Noll, H. Long, Y. Jin and W. Zhang, *Angewandte Chemie International Edition*, 2014, **53**, 10663-10667.
19. M. Mastalerz, M. W. Schneider, I. M. Oppel and O. Presly, *Angewandte Chemie International Edition*, 2011, **50**, 1046-1051.
20. T. Tozawa, J. T. A. Jones, S. I. Swamy, S. Jiang, D. J. Adams, S. Shakespeare, R. Clowes, D. Bradshaw, T. Hasell, S. Y. Chong, C. Tang, S. Thompson, J. Parker, A. Trewin, J. Bacsa, A. M. Z. Slawin, A. Steiner and A. I. Cooper, *Nature Materials*, 2009, **8**, 973-978.
21. H. Takahagi, S. Fujibe and N. Iwasawa, *Chemistry – A European Journal*, 2009, **15**, 13327-13330.
22. K. Ono, K. Johmoto, N. Yasuda, H. Uekusa, S. Fujii, M. Kiguchi and N. Iwasawa, *Journal of the American Chemical Society*, 2015, **137**, 7015-7018.
23. J. Tian, P. K. Thallapally, S. J. Dalgarno, P. B. McGrail and J. L. Atwood, *Angewandte Chemie International Edition*, 2009, **48**, 5492-5495.
24. B. Ser Park, C. B. Knobler and D. J. Cram, *Chemical Communications*, 1998, 55-56.

25. A. Avellaneda, P. Valente, A. Burgun, J. D. Evans, A. W. Markwell-Heys, D. Rankine, D. J. Nielsen, M. R. Hill, C. J. Sumby and C. J. Doonan, *Angewandte Chemie International Edition*, 2013, **52**, 3746-3749.
26. K. Harata, *Bulletin of the Chemical Society of Japan*, 1977, **50**, 1416-1424.
27. M. M. Ayhan, H. Karoui, M. Hardy, A. Rockenbauer, L. Charles, R. Rosas, K. Udachin, P. Tordo, D. Bardelang and O. Ouari, *Journal of the American Chemical Society*, 2015, **137**, 10238-10245.
28. T. Brotin, D. Cavagnat, E. Jeanneau and T. Buffeteau, *The Journal of Organic Chemistry*, 2013, **78**, 6143-6153.
29. D. Dubbeldam, S. Calero, D. E. Ellis and R. Q. Snurr, *Molecular Simulation*, 2016, **42**, 81-101.
30. S. L. Mayo, B. D. Olafson and W. A. Goddard III, *J. Phys. Chem.*, 1990, **94**, 8897-8909.
31. A. K. Rappe, C. J. Casewit, K. S. Colwell, W. A. Goddard III and W. M. Skiff, *Journal of American Chemical Society*, 1992, **114**, 10035-10046.
32. R. C. Reid, J. M. Prausnitz and B. E. Poling, *The properties of gases and liquids*, McGraw Hill Book Co.,New York, NY, United States, 1987.
33. Y. Kohyama, T. Murase and M. Fujita, *Angewandte Chemie International Edition*, 2014, **53**, 11510-11513.
34. H. Takezawa, T. Murase, G. Resnati, P. Metrangolo and M. Fujita, *Angewandte Chemie International Edition*, 2015, **54**, 8411-8414.
35. C. F. Macrae, I. Sovago, S. J. Cottrell, P. T. A. Galek, P. McCabe, E. Pidcock, M. Platings, G. P. Shields, J. S. Stevens, M. Towler and P. A. Wood, *Journal of Applied Crystallography*, 2020, **53**, 226-235.
36. J. M. Vicent-Luna, *SITES-ANALYZER*, https://github.com/jmviclun/SITES-ANALYZER, 2020.
